# Analysis of newly developed fractal-fractional derivative with power law kernel for MHD couple stress fluid in channel embedded in a porous medium

**DOI:** 10.1038/s41598-021-00163-3

**Published:** 2021-10-21

**Authors:** Muhammad Arif, Poom Kumam, Wiyada Kumam, Ali Akgul, Thana Sutthibutpong

**Affiliations:** 1 Fixed Point Research Laboratory, Fixed Point Theory and Applications Research Group, Center of Excellence in Theoretical and Computational Science (TaCS-CoE), Faculty of Science, King Mongkut’s University of Technology Thonburi (KMUTT), 126 Pracha Uthit Rd., Bang Mod, Thung Khru, Bangkok, 10140 Thailand; 2 Center of Excellence in Theoretical and Computational Science (TaCS-CoE), Faculty of Science, King Mongkut’s University of Technology Thonburi (KMUTT), 126 Pracha Uthit Rd., Bang Mod, Thung Khru, Bangkok, 10140 Thailand; 3 Department of Medical Research, China Medical University Hospital, China Medical University, Taichung, 40402 Taiwan; 4 Program in Applied Statistics, Department of Mathematics and Computer Science, Faculty of Science and Technology, Rajamangala University of Technology Thanyaburi (RMUTT), Pathum Thani, 12110 Thailand; 5 Department of Mathematics, Faculty of Arts and Sciences, Siirt University, TR-56100 Siirt, Turkey; 6 Theoretical and Computational Physics Group, Department of Physics, Faculty of Science, King Mongkut’s University of Technology Thonburi (KMUTT), 126 Pracha-Uthit Road, Bang Mod, Thung Khru, Bangkok 10140 Thailand

**Keywords:** Engineering, Mathematics and computing

## Abstract

Fractal-fractional derivative is a new class of fractional derivative with power Law kernel which has many applications in real world problems. This operator is used for the first time in such kind of fluid flow. The big advantage of this operator is that one can formulate models describing much better the systems with memory effects. Furthermore, in real world there are many problems where it is necessary to know that how much information the system carries. To explain the memory in a system fractal-fractional derivatives with power law kernel is analyzed in the present work. Keeping these motivation in mind in the present paper new concept of fractal-fractional derivative for the modeling of couple stress fluid (CSF) with the combined effect of heat and mass transfer have been used. The magnetohydrodynamics (MHD) flow of CSF is taken in channel with porous media in the presence of external pressure. The constant motion of the left plate generates the CSF motion while the right plate is kept stationary. The non-dimensional fractal-fractional model of couple stress fluid in Riemann–Liouville sense with power law is solved numerically by using the implicit finite difference method. The obtained solutions for the present problem have been shown through graphs. The effects of various parameters are shown through graphs on velocity, temperature and concentration fields. The velocity, temperature and concentration profiles of the MHD CSF in channel with porous media decreases for the greater values of both fractional parameter $\alpha$ and fractal parameter $\beta$ respectively. From the graphical results it can be noticed that the fractal-fractional solutions are more general as compared to classical and fractional solutions of CSF motion in channel. Furthermore, the fractal-fractional model of CSF explains good memory effect on the dynamics of couple stress fluid in channel as compared to fractional model of CSF. Finally, the skin friction, Nusselt number and Sherwood number are evaluated and presented in tabular form.

## Introduction

Fractional calculus is used to explain many real world problems with better memory effect. Fractional calculus FC is the generalization of integer order calculus which was not sufficient to explain some memory effect in some engineering and real world problems. Due to its enormous applications in many fields of sciences the FC is getting attention of the researchers from the last three decades. In other words FC is used to describe the historical states of different phenomenon which we call as memory. Many researchers developed different definitions of fractional derivatives for different physical situations. Some applications of FC are given in the Tenreiro et al*.*^1^. Dalir and Bashour^2^ investigated some applications of FC in applied sciences. Tavazoei et al*.*^3^ studied some uses of FC in modern sciences and in the processes of suppression of chaotic oscillations. Similarly, Sabatier et al*.*^4^ discussed some advanced applications of FC in modern sciences. Koca and Atangana^5^ investigated Cattaneo-Hristov model with the effect of heat diffusion using Caputo-Fabrizio and Atangana-Baleanu fractional derivatives. In another paper Atangana and Baleanu^6^ discussed new fractional derivatives to highlight some applications and theory of heat and mass transfer models. Arif et al*.*^7^ investigated CSF in channel. In this study they generalized the classical model of CSF into time fractional models of AB and CF and compare their results. Podlubny^8^ discussed fractional differential equations and their uses in different physical phenomenon. Abdeljawad and Baleanu^9^ studied fractional derivatives and their applications with generalized Mittag–Leffler function.

Recently, Atangana^10^ developed the new idea of fractal-fractional derivative in FC. This new idea is very suitable in many situations in dealing some complex problems. In the operator there are two orders, the first is known as fractional order and the second operator is called the fractal dimension. This new idea of fractal fractional derivative is better than the classical one and fractional derivatives as well. It is due to the fact that by dealing with fractal-fractional derivatives we can study the fractional operator as well as fractal dimension at the same time. Motivated from the advance and unique properties many researchers taking interest in fractal-fractional operator. Ali et al*.*^11^ investigated SIR model to study the dynamics of COVID-19 using fractal-fractional operator. Recently, Akgul and Siddique^12^ investigated the fluid flow in channel with MHD effect using the idea of fractal-fractional derivatives on the CSF. Esmonde^13^ studied fractal-fractional derivative modeling for the applications of material phase change. In another paper Akgul^14^ discussed some advance and new applications of fractal fractional differential equations using power law kernel.

Mainly, fluids are classified in two categories, Newtonian and non-Newtonian. Most of the real world problems are analyzed by non-Newtonian fluids. Many researchers have shown interest in non- Newtonian fluids due to enormous applications in daily life. Some applications of non-Newtonian fluids flow like laminar flow, micro channel flows and pipe flows are given in^15^. Some industrial and engineering applications of non-Newtonian fluids are given in the book of Chhabra and Richardson^16^. Pawar and Sunnapwar^17^ examined some experimental study of non-Newtonian fluids in helical coils with laminar and turbulent flow of the non-Newtonian fluids. The advanced applications of non-Newtonian fluids like, oil pipeline friction reduction, heating and cooling systems, scale-up and flow tracers are investigated by Hoyt^18^. Sohail et al*.*^19^ investigated the features of non-Newtonian fluid and thermophysical characteristics of yield exhibiting non-Newtonian fluid flow under gyrotactic microorganisms. Bao et al.^20^ discussed the applications and practical uses of non-Newtonian fluids and calculated numerical modeling for this fluid flow in fractures and porous media. Some other applications of non-Newtonian fluid are given in^21–23^.

Couple stress fluid CSF is in the class of non-Newtonian fluids and have many engineering applications in real world. The idea of couple stresses in fluid was first given by Stokes^24^. After stokes many researchers used the model of CSF in different situations. Arif et al.^25^ discussed CSF in channel using AB fractional derivatives with gold nanoparticles suspended in the blood. Researchers are taking interest to analyzed CSF fluid in different domain for different scientific reasons. Like Adesanya et al*.*^26^ discussed irreversibility process using CSF in inclined channel with isothermal boundaries. Krishna and Chamkha^27^ studied engineering applications using the concept of CSF through a porous media with slip effect. Khan et al*.*^28^ investigated some biomechanical problems of CSF with expanding or contracting porous channel. Akhtar et al*.*^29^ studied CSF in channel using fractional derivatives.

MHD play a vital role in different fluid flow problems and have many applications in biological sciences and engineering sciences. The applications of MHD is not limited to fluid flow problems but it can be used in many engineering problems like Kumar et al*.*^30^ discussed the impact of magnetic dipole on the fluid flow over a stretching cylinder. Gowda et al*.*^31^ investigated MHD effect in their study using magnetized flow with the theory of heat diffusion and Stefan blowing condition. In another paper Gowda et al*.*^32^ examined the contribution of MHD flow over a stretching sheet exploring the magnetic dipole on the fluid flow. Yusuf et al*.*^33^ discussed magneto-Bioconvection flow over an inclined plate with entropy generation. Kumar et al*.*^34^ discussed the impact of MHD on thermophoretic particle in the fluid flow over a stretching sheet.

Heat and mass transfer have useful applications in industries and different engineering problems. Researchers investigate the effect of heat and mass transfer in different circumstances like Naveen et al*.*^35^ examined the influence of heat and mass transfer on the fluid flow along a stretching cylinder. Similarly Punith et al*.*^36^ discussed the impact of binary chemical reaction with the combined effect of heat and mass transfer on the fluid flow problem. Similarly other researchers examined the applications of heat and mass transfer in different field of sciences which are given in^37–39^.

From the above mentioned literature no work is reported to investigate the MHD flow of CSF in channel with heat and mass transfer. The fluid is taken in channel with porous media. The left plate is moving with constant velocity which generates the fluid motion while the right plate is fixed. The main novelty of the present article is to apply the fractal-fractional derivatives on the classical model. Fractal-fractional derivative is a new class of fractional derivative with power Law kernel which has many applications in real world problems. This operator is used for the first time in such kind of fluid flow. The big advantage of this operator is that we can formulate models describing much better the systems with memory effects. Furthermore, in real world there are many problems where we need to know that how much information the system carries that is why need memory in a system which is explained by fractal-fractional derivatives with power law kernel. This distribution in memory effect in fractional derivatives follows a power-law distribution. Keeping these motivations in mind the classical model of CSF is transform to fractal-fractional model and the numerical solutions have been obtained via implicit finite difference method.

## Formulation of the problem

In the present study we have considered the incompressible unsteady MHD flow of CSF in channel. The couple stress fluid is taken in channel in the presence of external pressure with body couples. The fluid is taken between two plates and porous medium is considered. Initially, both plates and fluid were at rest after some time the left plate start moving with constant velocity due to which the fluid flow in channel and the right plate is fixed. The governing equations for the present flow regime are given below:

The continuity equation is given by^25,29,40^:

1 $$\nabla \cdot {\mathbf{\mathop{V}\limits^{\rightharpoonup} }}{ = 0,}$$

The momentum equation of the given problem is given by:

2 $$\rho \frac{{D{\mathbf{\mathop{V}\limits^{\rightharpoonup} }}}}{Dt} = - \nabla p - \mu \nabla \times \left( {\nabla \times {\mathbf{\mathop{V}\limits^{\rightharpoonup} }}} \right) - \eta \nabla \times \left( {\nabla \times \left( {\nabla \times \left( {\nabla \times {\mathbf{\mathop{V}\limits^{\rightharpoonup} }}} \right)} \right)} \right) + \rho \vec{b} + r,$$

The energy equation can be written as:

3 $$\rho c_{p} \frac{{\partial {\mathbf{\mathop{T}\limits^{\rightharpoonup} }}}}{\partial t} = k\nabla \times \nabla \times {\mathbf{\mathop{T}\limits^{\rightharpoonup} }},$$

The concentration equation can be written as:

4 $$\frac{{\partial {\mathbf{\mathop{C}\limits^{\rightharpoonup} }}}}{\partial t} = D\nabla \times \nabla \times {\mathbf{\mathop{C}\limits^{\rightharpoonup} }},$$

where in the momentum equation the term $\rho \overrightarrow {b}$ shows the body forces which can be expressed as:

5 $$\rho \mathop{b}\limits^{\rightharpoonup} = {\mathbf{\mathop{J}\limits^{\rightharpoonup} }} \times {\mathbf{\mathop{B}\limits^{\rightharpoonup} }} + \rho \mathop{g}\limits^{\rightharpoonup} .$$

Here ${\mathbf{\mathop{V}\limits^{\rightharpoonup} }},{\mathbf{\mathop{T}\limits^{\rightharpoonup} }}$ and ${\mathbf{\mathop{C}\limits^{\rightharpoonup} }}$ are the velocity, temperature and concentration vectors. $p,\rho ,\mu ,\rho \vec{b},k,D,\, \, \vec{g}\,\,{\text{and}}\,\,{\text{r}}$ is the pressure, density, dynamic viscosity, body forces, thermal conductivity, thermal diffusivity, gravitational acceleration and Darcy resistance respectively. ${\mathbf{\mathop{J}\limits^{\rightharpoonup} }}$ and ${\mathbf{\mathop{B}\limits^{\rightharpoonup} }}$ is the current density and total magnetic field. As we have considered unidirectional flow, therefore, velocity, temperature and concentration fields of the given flow are as:

6 $$\left. \begin{gathered} {\mathbf{\mathop{V}\limits^{\rightharpoonup} }} = \left( {u_{1} (y,t),0,0} \right), \hfill \\ {\mathbf{\mathop{T}\limits^{\rightharpoonup} }} = \left( {T(y,t),0,0} \right), \hfill \\ {\mathbf{\mathop{C}\limits^{\rightharpoonup} }} = \left( {C(y,t),0,0} \right). \hfill \\ \end{gathered} \right\}$$

In the given study we have consider Darcy resistance in CSF, therefor, Darcy’s law can be written in the following form:

7 $$- \frac{\mu \phi }{{k_{0} }}.\overrightarrow {V} = r,$$

where $\phi$ represent porous media and $k$ represent permeability of the porous medium. Maxwell equation can be defined as:

From Maxwell equation:

8 $$\overrightarrow {\nabla } \cdot {\varvec{B}} = 0,$$

9 $$\overrightarrow {\nabla } \times \overrightarrow {E} = - \frac{{\partial {\varvec{B}}}}{\partial E} = 0,{\text{Where }}E = 0,$$

here $E$ is the total electric field. By Ohm’s Law (generalized form):

10 $${\varvec{J}} = \sigma \left( {\overrightarrow {E} + \overrightarrow {V} \times {\varvec{B}}} \right) = \sigma \left( {\overrightarrow {V} \times {\varvec{B}}} \right).$$

Cross product with the magnetic field is:

11 $${\varvec{J}} \times {\varvec{B}} = \sigma \left( {\overrightarrow {V} \times {\varvec{B}}} \right) \times \user2{B, B} = B_{0} + b,$$

here $B_{0}$ is applied magnetic field and $b$ is induced magnetic field by polarization. Now applying the vector scalar triple product, Eq. (11) becomes:

12 $$\user2{J } \times {\varvec{B}} = - \sigma \left\{ {{\varvec{B}} \times \left( {\overrightarrow {V} \times {\varvec{B}}} \right)} \right\} = - \sigma \left\{ {\left( {{\varvec{B}} \cdot {\varvec{B}}} \right)\overrightarrow {V} - \left( {{\varvec{B}} \cdot \overrightarrow {V} } \right){\varvec{B}}} \right\}$$

13 $${\varvec{J}} \times {\varvec{B}} = - \sigma \left\{ {\left( {{\varvec{B}} \cdot {\varvec{B}}} \right)\overrightarrow {V} - 0} \right\} = - \sigma {\varvec{B}}_{0}^{2} u.$$

**Figure 1:**
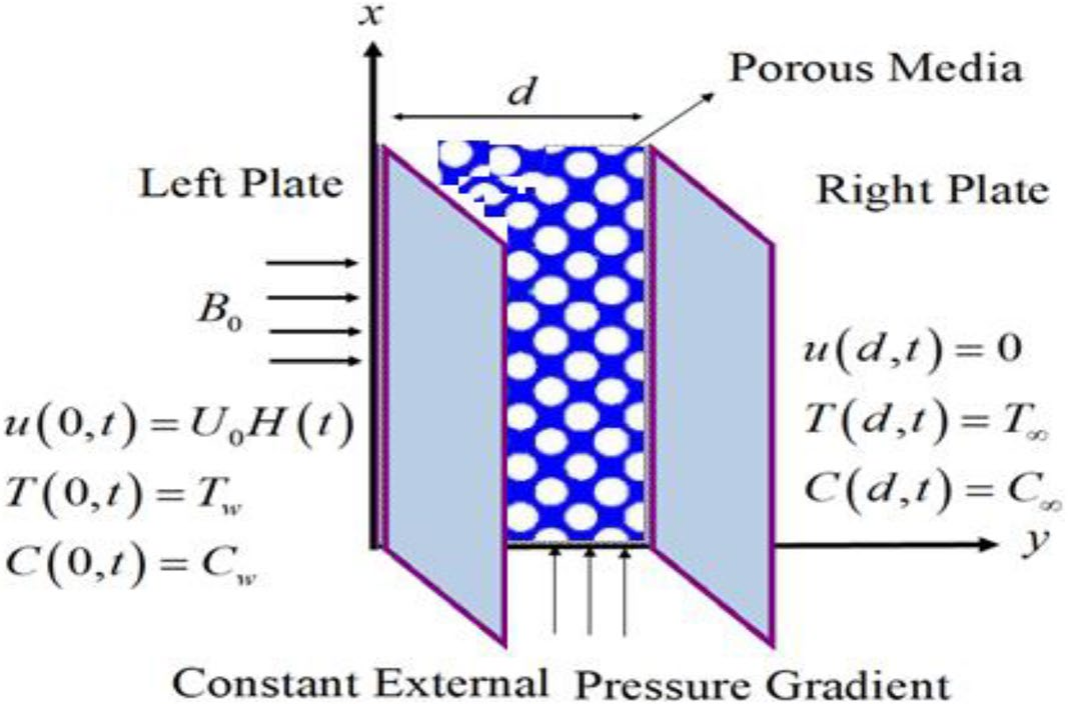
Geometry of the problem.

In this article we have considered the incompressible unsteady MHD flow of CSF in channel. The MHD CSF laminar fluid is considered to flow through an open channel of two parallel plates separated by a distance $d$*.* The medium is considered as a porous medium with porosity *K* in the presence of constant external pressure gradient *G* and the induced magnetic field *B*_*0*_ which is taken normal to the fluid flow. The motion of the fluid is considered in *x*-direction. Initially, for $t \le 0$ the fluid and both the plates are stationary with surrounding temperature $T_{\infty }$ and constant concentration $C_{\infty }$. At $t = 0^{ + }$, the temperature and concentration of the left plate raised to $T$ and $C$ respectively. As a result the left plate moving with constant velocity and the right plate is stationary. The geometrical sketch of the considered model is given in Fig. 1.

Using the assumptions which are considered in the problem the governing equations for the flow, energy and concentration equations are given by^2,25^:

14 $$\rho \frac{\partial u}{{\partial t}} = G + \mu \frac{{\partial^{2} u}}{{\partial y^{2} }} - \eta \frac{{\partial^{4} u}}{{\partial y^{4} }} - \sigma {\varvec{B}}_{0}^{2} u - \frac{\mu \phi }{{k_{0} }}u + g\rho \beta (T - T_{\infty } ) + g\rho \beta (C - C_{\infty } ),$$

15 $$\left( {\rho c_{p} } \right)\frac{{\partial T\left( {y,t} \right)}}{\partial t} = k\frac{{\partial^{2} T\left( {y,t} \right)}}{{\partial y^{2} }}$$

16 $$\frac{{\partial C\left( {y,t} \right)}}{\partial t} = D\frac{{\partial^{2} C\left( {y,t} \right)}}{{\partial y^{2} }}$$

with the physical initial and boundary conditions:

17 $$\left. \begin{gathered} u(y,0) = 0, \, T(y,0) = T_{\infty } , \, C(y,0) = C_{\infty } ,{\text{ for }}0 \le y \le d, \hfill \\ u(0,t) = H(t)U_{0} , \, T(0,t) = T_{w} , \, C(0,t) = C_{w} ,{\text{ for }}t > 0, \hfill \\ u(d,t) = 0, \, T(d,t) = T_{\infty } , \, C(d,t) = C_{\infty } ,{\text{ for }}t > 0, \hfill \\ \frac{{\partial^{2} u(0,t)}}{{\partial y^{2} }} = \frac{{\partial^{2} u(d,t)}}{{\partial y^{2} }} = 0,{\text{ for }}t > 0. \hfill \\ \end{gathered} \right\}$$

From the above initial and boundary conditions it can be observed that the fluid and plates were at rest initially. After some time the left plate $u(0,t) = H(t)U_{0}$ moving with constant velocity $U_{0}$, where $H(t)$ shows the Heaviside step function and the right plate is fixed. At the left plate the wall temperature and concentration and at the right plate there is ambient temeprature and concentration. Furthermore, $\frac{{\partial^{2} u(0,t)}}{{\partial y^{2} }} = \frac{{\partial^{2} u(d,t)}}{{\partial y^{2} }} = 0$, shows the couple shear stresses at the left and right plates.

For dimensional analysis, the following non-dimensional variables are introduced:

18 $$\left. \begin{gathered} \xi = \frac{y}{d},{\text{ w}} = \frac{u}{{U_{0} }}, \, \tau = \frac{{U_{0} t}}{d}, \, \eta_{A} = \frac{\eta }{{\mu d^{2} }}, \hfill \\ P = \frac{{d^{2} }}{{\mu U_{0} }}G, \, \Theta = \frac{{T - T_{\infty } }}{{T_{w} - T_{\infty } }}, \, \Phi = \frac{{C - C_{\infty } }}{{C_{w} - C_{\infty } }}, \hfill \\ \end{gathered} \right\}$$

After dimensionalization process we get the following dimensionless system of equations along with initial and boundary conditions.

19 $${\text{Re}} \frac{{\partial w\left( {\xi ,\tau } \right)}}{\partial \tau } = P + \frac{{\partial^{2} w\left( {\xi ,\tau } \right)}}{{\partial \xi^{2} }} - \eta_{A} \frac{{\partial^{4} w\left( {\xi ,\tau } \right)}}{{\partial \xi^{4} }} - Hw\left( {\xi ,\tau } \right) + Gr\Theta \left( {\xi ,\tau } \right) + Gm\Phi \left( {\xi ,\tau } \right),$$

20 $$A\frac{{\partial \Theta \left( {\xi ,\tau } \right)}}{\partial \tau } = \frac{{\partial^{2} \Theta \left( {\xi ,\tau } \right)}}{{\partial \xi^{2} }}$$

21 $$B\frac{{\partial \Phi \left( {\xi ,\tau } \right)}}{\partial \tau } = \frac{{\partial^{2} \Phi \left( {\xi ,\tau } \right)}}{{\partial \xi^{2} }}$$

22 $$\left. \begin{gathered} w\left( {\xi ,0} \right) = 0, \, \Theta \left( {\xi ,0} \right) = 0, \, \Phi \left( {\xi ,0} \right) = 0,{\text{ for }}0 \le \xi \le d \hfill \\ w\left( {0,\tau } \right) = 1, \, \Theta \left( {0,\tau } \right) = 1, \, \Phi \left( {0,\tau } \right) = 1,{\text{ for }}\tau > 0, \hfill \\ w\left( {d,\tau } \right) = 0, \, \Theta \left( {d,\tau } \right) = 0, \, \Phi \left( {d,\tau } \right) = 0,{\text{ for }}\tau > 0, \hfill \\ \frac{{\partial^{2} w\left( {0,\tau } \right)}}{{\partial \xi^{2} }} = \frac{{\partial^{2} w\left( {d,\tau } \right)}}{{\partial \xi^{2} }} = 0,{\text{ for }}\tau > 0, \hfill \\ \end{gathered} \right\}$$

$$Gr = \frac{{g\rho \beta_{T} d^{2} \left( {T_{w} - T_{\infty } } \right)}}{{U_{0} \mu }}, \, Gm = \frac{{g\rho \beta_{C} d^{2} \left( {C_{w} - C_{\infty } } \right)}}{{U_{0} \mu }},\,\,\Pr = \frac{{\mu c_{p} }}{k}, \, {\text{Re}} = \frac{{U_{0} d}}{\upsilon }, \, Sc = \frac{\upsilon }{D},$$

$$M = \frac{{\sigma {\varvec{B}}_{0}^{2} d^{2} }}{\mu },\,\frac{1}{K} = \frac{{d^{2} \phi }}{{k_{0} }},\,\,\,\,H = M + \frac{1}{K},\,\,A = \Pr .{\text{Re}} ,\,\,B = Sc.{\text{Re}} .$$

where $Gr$ represents Grashof number, $Gm$ mass Grashof number, $\Pr$ represents Prandtl number, ${\text{Re}}$ represents Reynolds number, $Sc$ represents Schmidth number, $M$ represnets magnetic parameter,$K$ porosity parameter and $H$ Hartmann number.

## Definition of Fractal-Fractional Derivative with Power Law Kernal

Let assume that $f(t)$ is continuous in the interval (a, b) and let the function is fractal differentiable on (a, b) having order $\beta$, then the fractal-fractional derivative of $f$ having order $\alpha$ in Riemann–Liouville RL sense with power law kernel is given by^10^:

23 $${}_{a}^{FFP} D_{t}^{\alpha ,\beta } f(t) = \frac{1}{\Gamma [n - \alpha ]}\frac{d}{{dt^{\beta } }}\int\limits_{a}^{t} {f\left( y \right)\left( {t - y} \right)^{n - \alpha - 1} dy,\,\,} n - 1 < \alpha \le n,\,\,\,\,0 < n - 1 < \beta \le n,$$

where

24 $$\frac{df\left( y \right)}{{dy^{\beta } }} = \mathop {\lim }\limits_{t \to y} \frac{f\left( t \right) - f\left( y \right)}{{t^{\beta } - y^{\beta } }}.$$

## Solutions of CSF with fractal-fractional derivative

In order to transform the classical CSF model into fractal-fractional derivative Eqs. (19–21) can be written in the following form:

25 $${}^{FFP}D_{\tau }^{\alpha ,\beta } w\left( {\xi ,\tau } \right) = \frac{1}{{\text{Re}}}\left( {P + \frac{{\partial^{2} w\left( {\xi ,\tau } \right)}}{{\partial \xi^{2} }} - \eta_{A} \frac{{\partial^{4} w\left( {\xi ,\tau } \right)}}{{\partial \xi^{4} }} - Hw\left( {\xi ,\tau } \right) + Gr\Theta \left( {\xi ,\tau } \right) + Gm\Phi \left( {\xi ,\tau } \right)} \right),$$

26 $${}^{FFP}D_{\tau }^{\alpha ,\beta } \Theta \left( {\xi ,\tau } \right) = \frac{1}{A}\left( {\frac{{\partial^{2} \Theta \left( {\xi ,\tau } \right)}}{{\partial \xi^{2} }}} \right),$$

27 $${}^{FFP}D_{\tau }^{\alpha ,\beta } \Phi \left( {\xi ,\tau } \right) = \frac{1}{B}\left( {\frac{{\partial^{2} \Phi \left( {\xi ,\tau } \right)}}{{\partial \xi^{2} }}} \right).$$

where ${}^{FFP}D_{t}^{\alpha ,\beta } (.,.)$ shows the fractal-fractional derivative, $0 < \alpha \le 1$ is the fractional order and $0 < \beta \le1$ is the fractal dimension.

### Solution of Energy Equation

From Eq. (26), the following result is obtained:

28 $${}^{FFP}D_{\tau }^{\alpha ,\beta } \Theta \left( {\xi ,\tau } \right) = \frac{1}{A}\left( {\frac{{\partial^{2} \Theta \left( {\xi ,\tau } \right)}}{{\partial \xi^{2} }}} \right),$$

Equation (28) can be written as:

29 $$\frac{1}{{\Gamma \left( {1 - \alpha } \right)}}.\frac{d}{d\tau }\int\limits_{0}^{\tau } {\Theta \left( {\xi ,t} \right)\left( {\tau - t} \right)^{ - \alpha } dt = \frac{{\beta .\tau^{\beta - 1} }}{A}} .\left( {\frac{{\partial^{2} \Theta \left( {\xi ,\tau } \right)}}{{\partial \xi^{2} }}} \right),$$

the above result can be written in the following form:

30 $$\frac{1}{{\Gamma \left( {1 - \alpha } \right)}}.\int\limits_{0}^{\tau } {\frac{{\partial \Theta \left( {\xi ,t} \right)}}{\partial t}.\left( {\tau - t} \right)^{ - \alpha } dt = \frac{{\beta .\tau^{\beta - 1} }}{A}} .\left( {\frac{{\partial^{2} \Theta \left( {\xi ,\tau } \right)}}{{\partial \xi^{2} }}} \right) - \frac{{\tau^{ - \alpha } \Theta \left( {\xi ,0} \right)}}{{\Gamma \left( {1 - \alpha } \right)}},$$

using the given initial condition from Eq. (22), Eq. (30) reduces to the following form:

31 $$\frac{1}{{\Gamma \left( {1 - \alpha } \right)}}.\int\limits_{0}^{\tau } {\frac{{\partial \Theta \left( {\xi ,t} \right)}}{\partial t}.\left( {\tau - t} \right)^{ - \alpha } dt = \frac{{\beta .\tau^{\beta - 1} }}{A}} .\left( {\frac{{\partial^{2} \Theta \left( {\xi ,\tau } \right)}}{{\partial \xi^{2} }}} \right),$$

by discretizing the above equation at $\left( {\xi_{i} ,\,\tau = \tau_{n + 1} } \right),$ the following form is obtained:

32 $$\frac{1}{{\Gamma \left( {1 - \alpha } \right)}}.\frac{d}{d\tau }\int\limits_{0}^{{\tau_{n + 1} }} {\frac{{\partial \Theta \left( {\xi_{i} ,t} \right)}}{\partial t}\left( {\tau_{n + 1} - t} \right)^{ - \alpha } dt = \frac{{\beta .\tau_{n + 1}^{\beta - 1} }}{A}} .\left( {\frac{{\Theta_{i + 1}^{n + 1} - 2\Theta_{i}^{n + 1} + \Theta_{i - 1}^{n + 1} }}{{\left( {\Delta \xi } \right)^{2} }}} \right),$$

From the above equation the following results are obtained:

33 $$\frac{1}{{\Gamma \left( {1 - \alpha } \right)}}.\sum\limits_{j = 0}^{n} {\frac{{\Theta_{i}^{j + 1} - 2\Theta_{i}^{j} }}{\Delta \tau }} .\int\limits_{{\tau_{j} }}^{{\tau_{j + 1} }} {\left( {\tau_{n + 1} - t} \right)^{ - \alpha } dt = \frac{{\beta .\tau_{n + 1}^{\beta - 1} }}{A}} .\left( {\frac{{\Theta_{i + 1}^{n + 1} - 2\Theta_{i}^{n + 1} + \Theta_{i - 1}^{n + 1} }}{{\left( {\Delta \xi } \right)^{2} }}} \right),$$

34 $$\frac{{\left( {\Delta \tau } \right)^{ - \alpha } }}{{\Gamma \left( {2 - \alpha } \right)}}.\sum\limits_{j = 0}^{n} {\frac{{\Theta_{i}^{j + 1} - 2\Theta_{i}^{j} }}{\Delta \tau }} .\left( {\left( {n - j + 1} \right)^{1 - \alpha } - \left( {n - j} \right)^{1 - \alpha } } \right) = \frac{\beta }{A}\left( {\left( {n + 1} \right)\Delta \tau } \right)^{\beta - 1} \left( {\frac{{\Theta_{i + 1}^{n + 1} - 2\Theta_{i}^{n + 1} + \Theta_{i - 1}^{n + 1} }}{{\left( {\Delta \xi } \right)^{2} }}} \right).$$

### Solution of concentration equation

From equation (27), the following result is obtained:

35 $${}^{FFP}D_{\tau }^{\alpha ,\beta } \Phi \left( {\xi ,\tau } \right) = \frac{1}{B}\left( {\frac{{\partial^{2} \Phi \left( {\xi ,\tau } \right)}}{{\partial \xi^{2} }}} \right),$$

Equation (35) can be written as:

36 $$\frac{1}{{\Gamma \left( {1 - \alpha } \right)}}.\frac{d}{d\tau }\int\limits_{0}^{\tau } {\Phi \left( {\xi ,t} \right)\left( {\tau - t} \right)^{ - \alpha } dt = \frac{{\beta .\tau^{\beta - 1} }}{B}} .\left( {\frac{{\partial^{2} \Phi \left( {\xi ,\tau } \right)}}{{\partial \xi^{2} }}} \right),$$

here

37 $$\frac{1}{{\Gamma \left( {1 - \alpha } \right)}}.\int\limits_{0}^{\tau } {\frac{{\partial \Phi \left( {\xi ,t} \right)}}{\partial t}.\left( {\tau - t} \right)^{ - \alpha } dt = \frac{{\beta .\tau^{\beta - 1} }}{B}} .\left( {\frac{{\partial^{2} \Phi \left( {\xi ,\tau } \right)}}{{\partial \xi^{2} }}} \right) - \frac{{\tau^{ - \alpha } \Phi \left( {\xi ,0} \right)}}{{\Gamma \left( {1 - \alpha } \right)}},$$

using the given initial condition from Eq. (22), Eq. (37) reduces to the following form:

38 $$\frac{1}{{\Gamma \left( {1 - \alpha } \right)}}.\int\limits_{0}^{\tau } {\frac{{\partial \Phi \left( {\xi ,t} \right)}}{\partial t}.\left( {\tau - t} \right)^{ - \alpha } dt = \frac{{\beta .\tau^{\beta - 1} }}{B}} .\left( {\frac{{\partial^{2} \Phi \left( {\xi ,\tau } \right)}}{{\partial \xi^{2} }}} \right),$$

by discretize the above equation at $\left( {\xi_{i} ,\,\tau = \tau_{n + 1} } \right),$ one can get the following result:

39 $$\frac{1}{{\Gamma \left( {1 - \alpha } \right)}}.\frac{d}{d\tau }\int\limits_{0}^{{\tau_{n + 1} }} {\frac{{\partial \Phi \left( {\xi_{i} ,t} \right)}}{\partial t}\left( {\tau_{n + 1} - t} \right)^{ - \alpha } dt = \frac{{\beta .\tau_{n + 1}^{\beta - 1} }}{B}} .\left( {\frac{{\Phi_{i + 1}^{n + 1} - 2\Phi_{i}^{n + 1} + \Phi_{i - 1}^{n + 1} }}{{\left( {\Delta \xi } \right)^{2} }}} \right),$$

the above result can be written as:

40 $$\frac{1}{{\Gamma \left( {1 - \alpha } \right)}}.\sum\limits_{j = 0}^{n} {\frac{{\Phi_{i}^{j + 1} - 2\Phi_{i}^{j} }}{\Delta \tau }} .\int\limits_{{\tau_{j} }}^{{\tau_{j + 1} }} {\left( {\tau_{n + 1} - t} \right)^{ - \alpha } dt = \frac{{\beta .\tau_{n + 1}^{\beta - 1} }}{B}} .\left( {\frac{{\Phi_{i + 1}^{n + 1} - 2\Phi_{i}^{n + 1} + \Phi_{i - 1}^{n + 1} }}{{\left( {\Delta \xi } \right)^{2} }}} \right),$$

41 $$\frac{{\left( {\Delta \tau } \right)^{ - \alpha } }}{{\Gamma \left( {2 - \alpha } \right)}}.\sum\limits_{j = 0}^{n} {\frac{{\Phi_{i}^{j + 1} - 2\Phi_{i}^{j} }}{\Delta \tau }} .\left( {\left( {n - j + 1} \right)^{1 - \alpha } - \left( {n - j} \right)^{1 - \alpha } } \right) = \frac{\beta }{B}\left( {\left( {n + 1} \right)\Delta \tau } \right)^{\beta - 1} \left( {\frac{{\Phi_{i + 1}^{n + 1} - 2\Phi_{i}^{n + 1} + \Phi_{i - 1}^{n + 1} }}{{\left( {\Delta \xi } \right)^{2} }}} \right).$$

## Solution of momentum equation

From Eq. (25), one can get the following fractal-fractional form of momentum equation:

42 $${}^{FFP}D_{\tau }^{\alpha ,\beta } w\left( {\xi ,\tau } \right) = \frac{1}{{\text{Re}}}\left( {P + \frac{{\partial^{2} w\left( {\xi ,\tau } \right)}}{{\partial \xi^{2} }} - \eta_{A} \frac{{\partial^{4} w\left( {\xi ,\tau } \right)}}{{\partial \xi^{4} }} - Hw\left( {\xi ,\tau } \right) + Gr\Theta \left( {\xi ,\tau } \right) + Gm\Phi \left( {\xi ,\tau } \right)} \right),$$

Equation (42) can be written as:

43 $$\frac{1}{{\Gamma \left( {1 - \alpha } \right)}}.\frac{d}{d\tau }\int\limits_{0}^{\tau } {w\left( {\xi ,\tau } \right)\left( {\tau - t} \right)^{ - \alpha } dt = \frac{{\beta .\tau^{\beta - 1} }}{{\text{Re}}}} .\left( \begin{gathered} P + \frac{{\partial^{2} w\left( {\xi ,\tau } \right)}}{{\partial \xi^{2} }} - \eta_{A} \frac{{\partial^{4} w\left( {\xi ,\tau } \right)}}{{\partial \xi^{4} }} \hfill \\ - Hw\left( {\xi ,\tau } \right) + Gr\Theta \left( {\xi ,\tau } \right) + Gm\Phi \left( {\xi ,\tau } \right) \hfill \\ \end{gathered} \right),$$

here

44 $$\frac{1}{{\Gamma \left( {1 - \alpha } \right)}}.\int\limits_{0}^{\tau } {\frac{{\partial w\left( {\xi ,t} \right)}}{\partial t}.\left( {\tau - t} \right)^{ - \alpha } dt = \frac{{\beta .\tau^{\beta - 1} }}{{\text{Re}}}} .\left( \begin{gathered} P + \frac{{\partial^{2} w\left( {\xi ,\tau } \right)}}{{\partial \xi^{2} }} \hfill \\ - \eta_{A} \frac{{\partial^{4} w\left( {\xi ,\tau } \right)}}{{\partial \xi^{4} }} - Hw\left( {\xi ,\tau } \right) \hfill \\ + Gr\Theta \left( {\xi ,\tau } \right) + Gm\Phi \left( {\xi ,\tau } \right) \hfill \\ \end{gathered} \right) - \frac{{\tau^{ - \alpha } w\left( {\xi ,0} \right)}}{{\Gamma \left( {1 - \alpha } \right)}},$$

using the given initial condition from Eq. (22), Eq. (44) reduces to the following form:

45 $$\frac{1}{{\Gamma \left( {1 - \alpha } \right)}}.\int\limits_{0}^{\tau } {\frac{{\partial w\left( {\xi ,t} \right)}}{\partial t}.\left( {\tau - t} \right)^{ - \alpha } dt = \frac{{\beta .\tau^{\beta - 1} }}{{\text{Re}}}} .\left( \begin{gathered} P + \frac{{\partial^{2} w\left( {\xi ,\tau } \right)}}{{\partial \xi^{2} }} \hfill \\ - \eta_{A} \frac{{\partial^{4} w\left( {\xi ,\tau } \right)}}{{\partial \xi^{4} }} - Hw\left( {\xi ,\tau } \right) \hfill \\ + Gr\Theta \left( {\xi ,\tau } \right) + Gm\Phi \left( {\xi ,\tau } \right) \hfill \\ \end{gathered} \right),$$

by discretizing the above equation at $\left( {\xi_{i} ,\,\tau = \tau_{n + 1} } \right),$ the discretized form is given as under:

46 $$\frac{1}{{\Gamma \left( {1 - \alpha } \right)}}.\int\limits_{0}^{{\tau_{n + 1} }} {\frac{{\partial w\left( {\xi_{i} ,t} \right)}}{\partial t}.\left( {\tau_{n + 1} - t} \right)^{ - \alpha } dt = \frac{{\beta .\tau_{n + 1}^{\beta - 1} }}{{\text{Re}}}} .\left( \begin{gathered} P + \frac{{w_{i + 1}^{n + 1} - 2w_{i}^{n + 1} + w_{i - 1}^{n + 1} }}{{\left( {\Delta \xi } \right)^{2} }} \hfill \\ - \eta_{A} \frac{{w_{i + 2}^{n + 1} - 4w_{i + 1}^{n + 1} + 6w_{i}^{n + 1} - 4w_{i - 1}^{n + 1} + w_{i - 2}^{n + 1} }}{{\left( {\Delta \xi } \right)^{4} }} \hfill \\ - Hw_{i}^{n + 1} \left( {\xi_{i} ,\tau_{n + 1} } \right) + Gr\Theta_{i}^{n + 1} \left( {\xi_{i} ,\tau_{n + 1} } \right) \hfill \\ + Gm\Phi_{i}^{n + 1} \left( {\xi_{i} ,\tau_{n + 1} } \right) \hfill \\ \end{gathered} \right),$$

from the above step the following result is obtained:

47 $$\frac{1}{{\Gamma \left( {1 - \alpha } \right)}}.\sum\limits_{j = 0}^{n} {\frac{{w_{i}^{j + 1} - 2w_{i}^{j} }}{\Delta \tau }} .\int\limits_{{\tau_{j} }}^{{\tau_{j + 1} }} {\left( {\tau_{n + 1} - t} \right)^{ - \alpha } dt = \frac{{\beta .\tau_{n + 1}^{\beta - 1} }}{{\text{Re}}}} .\left( \begin{gathered} P + \frac{{w_{i + 1}^{n + 1} - 2w_{i}^{n + 1} + w_{i - 1}^{n + 1} }}{{\left( {\Delta \xi } \right)^{2} }} \hfill \\ - \eta_{A} \frac{{w_{i + 2}^{n + 1} - 4w_{i + 1}^{n + 1} + 6w_{i}^{n + 1} - 4w_{i - 1}^{n + 1} + w_{i - 2}^{n + 1} }}{{\left( {\Delta \xi } \right)^{4} }} \hfill \\ - Hw_{i}^{n + 1} \left( {\xi_{i} ,\tau_{n + 1} } \right) + Gr\Theta_{i}^{n + 1} \left( {\xi_{i} ,\tau_{n + 1} } \right) \hfill \\ + Gm\Phi_{i}^{n + 1} \left( {\xi_{i} ,\tau_{n + 1} } \right) \hfill \\ \end{gathered} \right),$$

48 $$\begin{gathered} \frac{{\left( {\Delta \tau } \right)^{ - \alpha } }}{{\Gamma \left( {2 - \alpha } \right)}}.\sum\limits_{j = 0}^{n} {\frac{{w_{i}^{j + 1} - 2w_{i}^{j} }}{\Delta \tau }} .\left( {\left( {n - j + 1} \right)^{1 - \alpha } - \left( {n - j} \right)^{1 - \alpha } } \right) \hfill \\ = \frac{\beta }{{\text{Re}}}\left( {\left( {n + 1} \right)\Delta \tau } \right)^{\beta - 1} \left( \begin{gathered} P + \frac{{w_{i + 1}^{n + 1} - 2w_{i}^{n + 1} + w_{i - 1}^{n + 1} }}{{\left( {\Delta \xi } \right)^{2} }} - \eta_{A} \frac{{w_{i + 2}^{n + 1} - 4w_{i + 1}^{n + 1} + 6w_{i}^{n + 1} - 4w_{i - 1}^{n + 1} + w_{i - 2}^{n + 1} }}{{\left( {\Delta \xi } \right)^{4} }} \hfill \\ - Hw_{i}^{n + 1} \left( {\xi_{i} ,\tau_{n + 1} } \right) + Gr\Theta_{i}^{n + 1} \left( {\xi_{i} ,\tau_{n + 1} } \right) + Gm\Phi_{i}^{n + 1} \left( {\xi_{i} ,\tau_{n + 1} } \right) \hfill \\ \end{gathered} \right). \hfill \\ \end{gathered}$$

## Limiting case

In this section the present obtained solutions are reduced to already published work in order to verify the obtained solutions. Therefore, by putting $$\left( {Gr = 0} \right),$$$$\left( {Gm = 0} \right),$$$$P = 0$$ and $$\frac{1}{K} \to 0$$ present solutions reduced to the solutions recently obtained by Akgül and Siddique^12^ which verify the present results.

Using the above assumptions Eq. (25) reduces to the following form:

49 $${}^{C}D_{\tau }^{\alpha ,\beta } w\left( {\xi ,\tau } \right) = \frac{1}{{\text{Re}}}\left( {\frac{{\partial^{2} w\left( {\xi ,\tau } \right)}}{{\partial \xi^{2} }} - \eta_{A} \frac{{\partial^{4} w\left( {\xi ,\tau } \right)}}{{\partial \xi^{4} }} - Mw\left( {\xi ,\tau } \right)} \right),$$

Equation (49) can be written as:

50 $$\frac{1}{{\Gamma \left( {1 - \alpha } \right)}}.\frac{d}{d\tau }\int\limits_{0}^{\tau } {w\left( {\xi ,\tau } \right)\left( {\tau - t} \right)^{ - \alpha } dt = \frac{{\beta .\tau^{\beta - 1} }}{{\text{Re}}}} .\left( {\frac{{\partial^{2} w\left( {\xi ,\tau } \right)}}{{\partial \xi^{2} }} - \eta_{A} \frac{{\partial^{4} w\left( {\xi ,\tau } \right)}}{{\partial \xi^{4} }} - Mw\left( {\xi ,\tau } \right)} \right),$$

here

51 $$\frac{1}{{\Gamma \left( {1 - \alpha } \right)}}.\int\limits_{0}^{\tau } {\frac{{\partial w\left( {\xi ,t} \right)}}{\partial t}.\left( {\tau - t} \right)^{ - \alpha } dt = \frac{{\beta .\tau^{\beta - 1} }}{{\text{Re}}}} .\left( \begin{gathered} \frac{{\partial^{2} w\left( {\xi ,\tau } \right)}}{{\partial \xi^{2} }} - \eta_{A} \frac{{\partial^{4} w\left( {\xi ,\tau } \right)}}{{\partial \xi^{4} }} \hfill \\ - Mw\left( {\xi ,\tau } \right) \hfill \\ \end{gathered} \right) - \frac{{\tau^{ - \alpha } w\left( {\xi ,0} \right)}}{{\Gamma \left( {1 - \alpha } \right)}},$$

using the given initial condition from Eq. (22), Eq. (51) gives to the following form:

52 $$\frac{1}{{\Gamma \left( {1 - \alpha } \right)}}.\int\limits_{0}^{\tau } {\frac{{\partial w\left( {\xi ,t} \right)}}{\partial t}.\left( {\tau - t} \right)^{ - \alpha } dt = \frac{{\beta .\tau^{\beta - 1} }}{{\text{Re}}}} .\left( {\frac{{\partial^{2} w\left( {\xi ,\tau } \right)}}{{\partial \xi^{2} }} - \eta_{A} \frac{{\partial^{4} w\left( {\xi ,\tau } \right)}}{{\partial \xi^{4} }} - Mw\left( {\xi ,\tau } \right)} \right),$$

by discretizing the above equation at $\left( {\xi_{i} ,\,\tau = \tau_{n + 1} } \right),$ one can get the following form:

53 $$\frac{1}{{\Gamma \left( {1 - \alpha } \right)}}.\int\limits_{0}^{{\tau_{n + 1} }} {\frac{{\partial w\left( {\xi_{i} ,t} \right)}}{\partial t}.\left( {\tau_{n + 1} - t} \right)^{ - \alpha } dt = \frac{{\beta .\tau_{n + 1}^{\beta - 1} }}{{\text{Re}}}} .\left( \begin{gathered} \frac{{w_{i + 1}^{n + 1} - 2w_{i}^{n + 1} + w_{i - 1}^{n + 1} }}{{\left( {\Delta \xi } \right)^{2} }} \hfill \\ - \eta_{A} \frac{{w_{i + 2}^{n + 1} - 4w_{i + 1}^{n + 1} + 6w_{i}^{n + 1} - 4w_{i - 1}^{n + 1} + w_{i - 2}^{n + 1} }}{{\left( {\Delta \xi } \right)^{4} }} \hfill \\ - Mw_{i}^{n + 1} \left( {\xi_{i} ,\tau_{n + 1} } \right) \hfill \\ \end{gathered} \right),$$

From the above equation the following result is obtaiend:

54 $$\frac{1}{{\Gamma \left( {1 - \alpha } \right)}}.\sum\limits_{j = 0}^{n} {\frac{{w_{i}^{j + 1} - 2w_{i}^{j} }}{\Delta \tau }} .\int\limits_{{\tau_{j} }}^{{\tau_{j + 1} }} {\left( {\tau_{n + 1} - t} \right)^{ - \alpha } dt = \frac{{\beta .\tau_{n + 1}^{\beta - 1} }}{{\text{Re}}}} .\left( \begin{gathered} \frac{{w_{i + 1}^{n + 1} - 2w_{i}^{n + 1} + w_{i - 1}^{n + 1} }}{{\left( {\Delta \xi } \right)^{2} }} \hfill \\ - \eta_{A} \frac{{w_{i + 2}^{n + 1} - 4w_{i + 1}^{n + 1} + 6w_{i}^{n + 1} - 4w_{i - 1}^{n + 1} + w_{i - 2}^{n + 1} }}{{\left( {\Delta \xi } \right)^{4} }} \hfill \\ - Mw_{i}^{n + 1} \left( {\xi_{i} ,\tau_{n + 1} } \right) \hfill \\ \end{gathered} \right),$$

55 $$\begin{gathered} \frac{{\left( {\Delta \tau } \right)^{ - \alpha } }}{{\Gamma \left( {2 - \alpha } \right)}}.\sum\limits_{j = 0}^{n} {\frac{{w_{i}^{j + 1} - 2w_{i}^{j} }}{\Delta \tau }} .\left( {\left( {n - j + 1} \right)^{1 - \alpha } - \left( {n - j} \right)^{1 - \alpha } } \right) \hfill \\ = \frac{\beta }{{\text{Re}}}\left( {\left( {n + 1} \right)\Delta \tau } \right)^{\beta - 1} \left( \begin{gathered} \frac{{w_{i + 1}^{n + 1} - 2w_{i}^{n + 1} + w_{i - 1}^{n + 1} }}{{\left( {\Delta \xi } \right)^{2} }} \hfill \\ - \eta_{A} \frac{{w_{i + 2}^{n + 1} - 4w_{i + 1}^{n + 1} + 6w_{i}^{n + 1} - 4w_{i - 1}^{n + 1} + w_{i - 2}^{n + 1} }}{{\left( {\Delta \xi } \right)^{4} }} \hfill \\ - Mw_{i}^{n + 1} \left( {\xi_{i} ,\tau_{n + 1} } \right) + \hfill \\ \end{gathered} \right). \hfill \\ \end{gathered}$$

## Nusselt number, Sherwood number and Skin friction:

### Nusselt number

Mathematically, Nusselt number for CSF can be written as:

56 $$Nu = - \left. {\frac{\partial \Theta }{{\partial \xi }}} \right|_{\xi = 0}$$

### Sherwood number:

Mathematically, Sherwood number for CSF can be written as:

57 $$Sh = - \left. {\frac{\partial \Phi }{{\partial \xi }}} \right|_{\xi = 0}$$

### Skin friction

Skin friction for CSF is as under:

58 $$Sf\left( {\xi ,\tau } \right) = \left( {\frac{\partial w}{{\partial \xi }} - \eta \frac{{\partial^{3} w}}{{\partial \xi^{3} }}} \right)$$

As the given flow model is between two parallel plates. Therefore, the skin friction at the left and right plates is as under:

59 $$Sf_{lp} \left( {0,\tau } \right) = \left( {\frac{\partial w}{{\partial \xi }} - \eta \frac{{\partial^{3} w}}{{\partial \xi^{3} }}} \right)_{\xi = 0}$$

60 $$Sf_{rp} \left( {1,\tau } \right) = \left( {\frac{\partial w}{{\partial \xi }} - \eta \frac{{\partial^{3} w}}{{\partial \xi^{3} }}} \right)_{\xi = 1}$$

where $Sf_{lp} (.)$ and $Sf_{rp} (.)$ denotes the skin friction at left and right plates respectively.

## Results and discussion

This section provide fractal-fractional derivative model of unsteady MHD generalized Couette flow of CSF in channel with embedded in porous media with power law kernel. Numerical solutions for the proposed problem are obtained using the implicit finite difference method. In this study we have found the influence of fractal dimension and fractional operator on the fluid motion, fluid temeprature and concentration graphically. Furthermore, for clear understanding the influence of all parameters is shown through graphs which effect the fluid motion, temperature and concentration.

**Figure 2:**
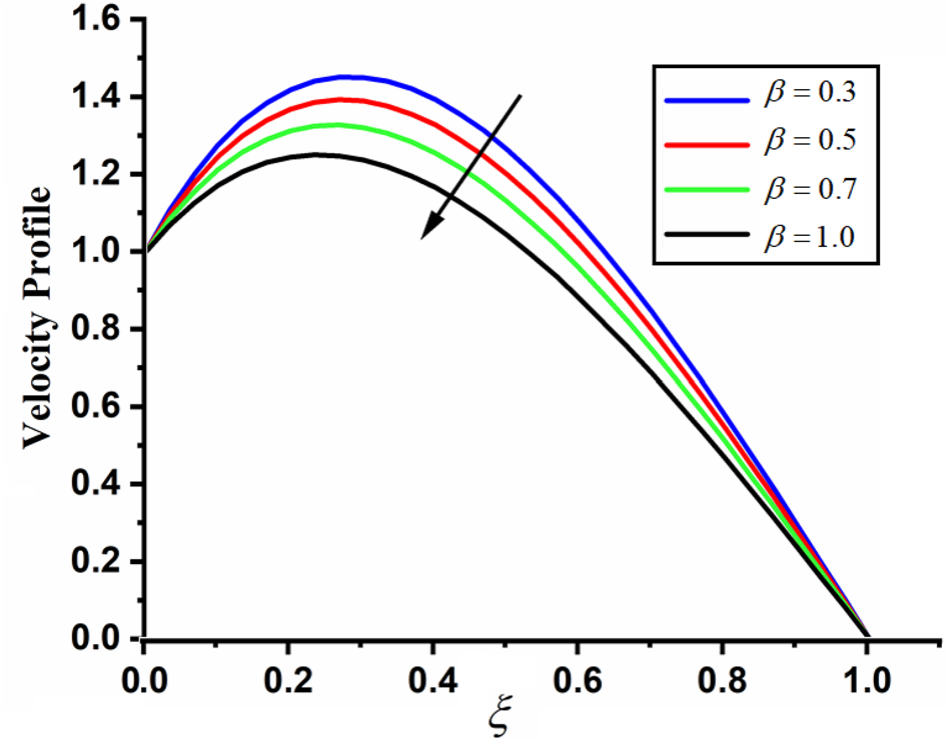
The impact of fractal parameter $\beta$ on couple stress fluid velocity in channel when $\alpha = 0.5$, $Gr = 2$, $Gm = 3$, $M = 2$, $K = 3$, $\eta_{A} = 1$, $P = 1.5$, $\Pr = 1.5$, ${\text{Re}} = 2.5$ and $Sc = 0.3$.

**Figure 3:**
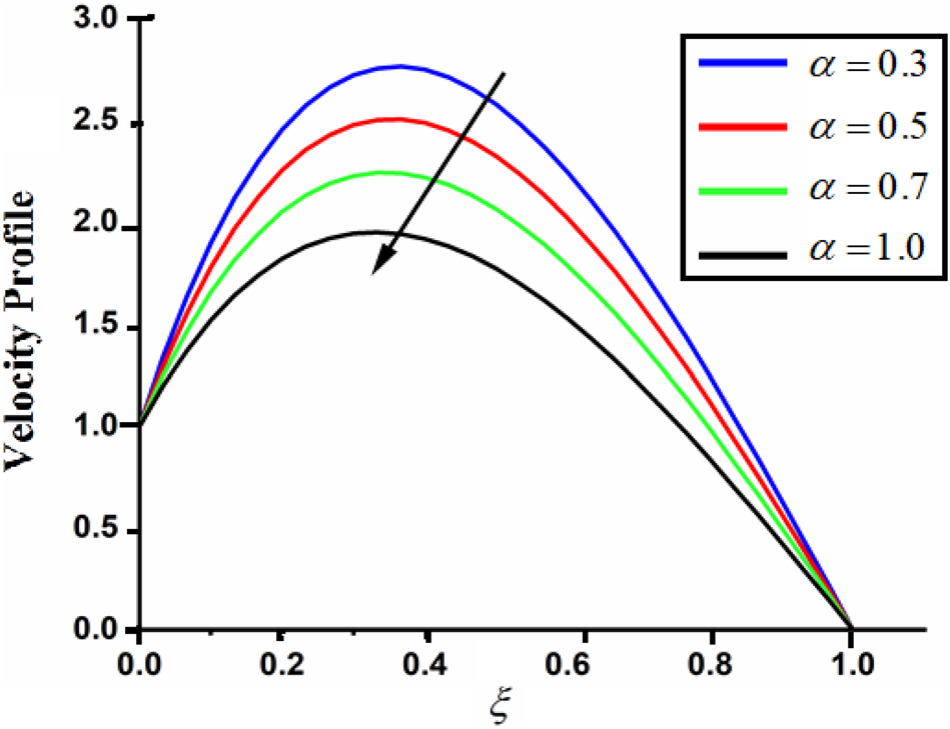
The impact of fractional parameter $\alpha$ on couple stress fluid velocity in channel when, $\beta = 0.5$, $Gr = 2$, $Gm = 3$, $M = 2$, $K = 3$, $\eta_{A} = 1$, $P = 1.5$, $\Pr = 1.5$, ${\text{Re}} = 2.5$ and $Sc = 0.3$.

**Figure 4:**
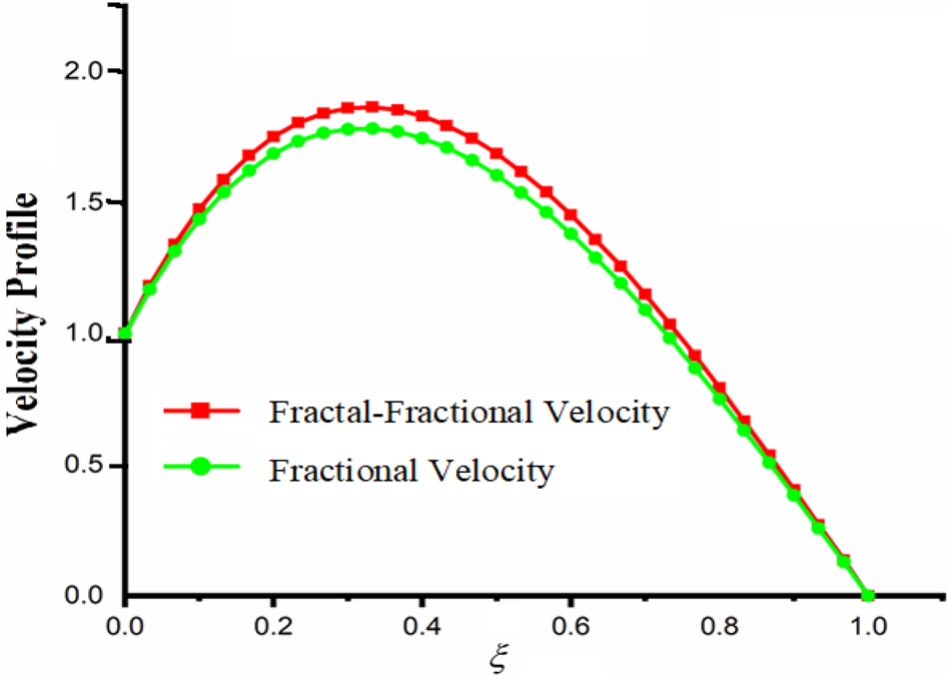
The comparison of fractal-fractional CSF velocity with fractional CSF velocity in channel when, $\alpha = 0.5$, $\beta = 0.5$, $Gr = 2$, $Gm = 3$, $M = 2$, $K = 3$, $\eta_{A} = 1$, $P = 1.5$, $\Pr = 1.5$, ${\text{Re}} = 2.5$ and $Sc = 0.3$.

**Figure 5:**
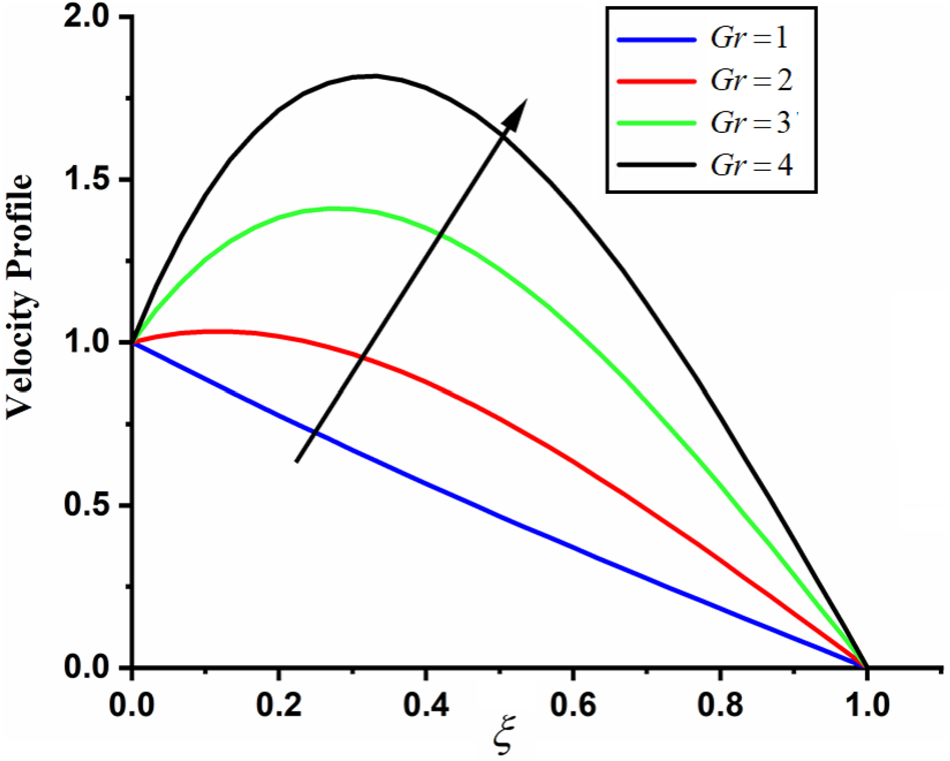
The impact of $Gr$ on fractal-fractional couple stress fluid velocity in channel when, $\alpha = 0.5$, $\beta = 0.5$, $Gm = 3$, $M = 2$, $K = 3$, $\eta_{A} = 1$, $P = 1.5$, $\Pr = 1.5$, ${\text{Re}} = 2.5$ and $Sc = 0.3$.

**Figure 6:**
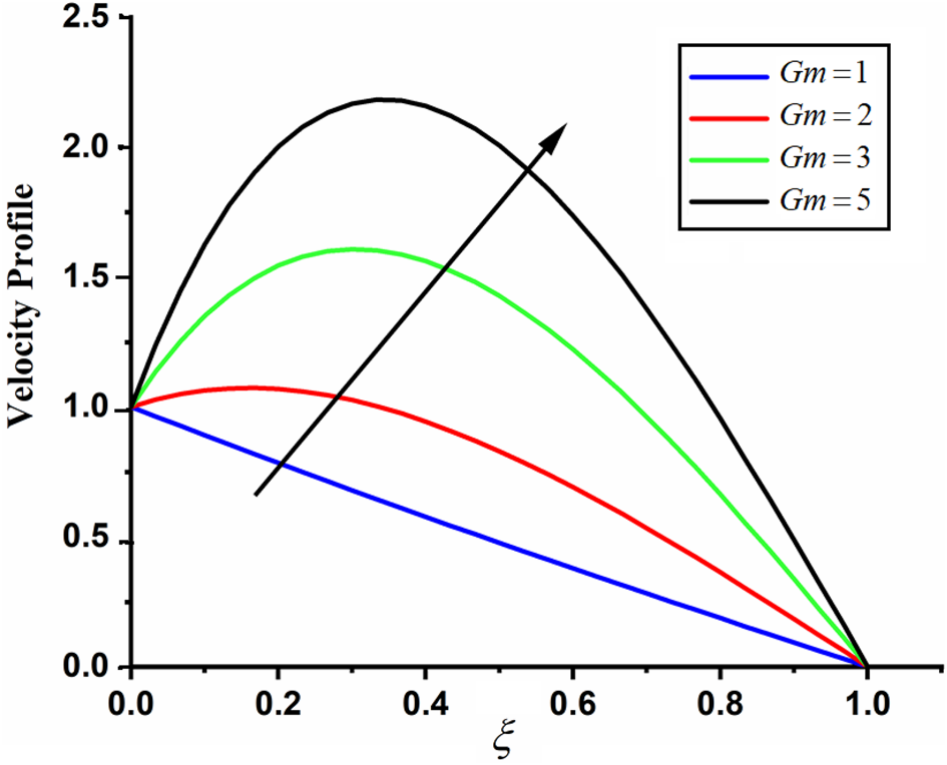
The impact of $Gm$ on fractal-fractional couple stress fluid velocity in channel when, $\alpha = 0.5$, $\beta = 0.5$, $Gr = 2$, $M = 2$, $K = 3$, $\eta_{A} = 1$, $P = 1.5$, $\Pr = 1.5$, ${\text{Re}} = 2.5$ and $Sc = 0.3$.

**Figure 7:**
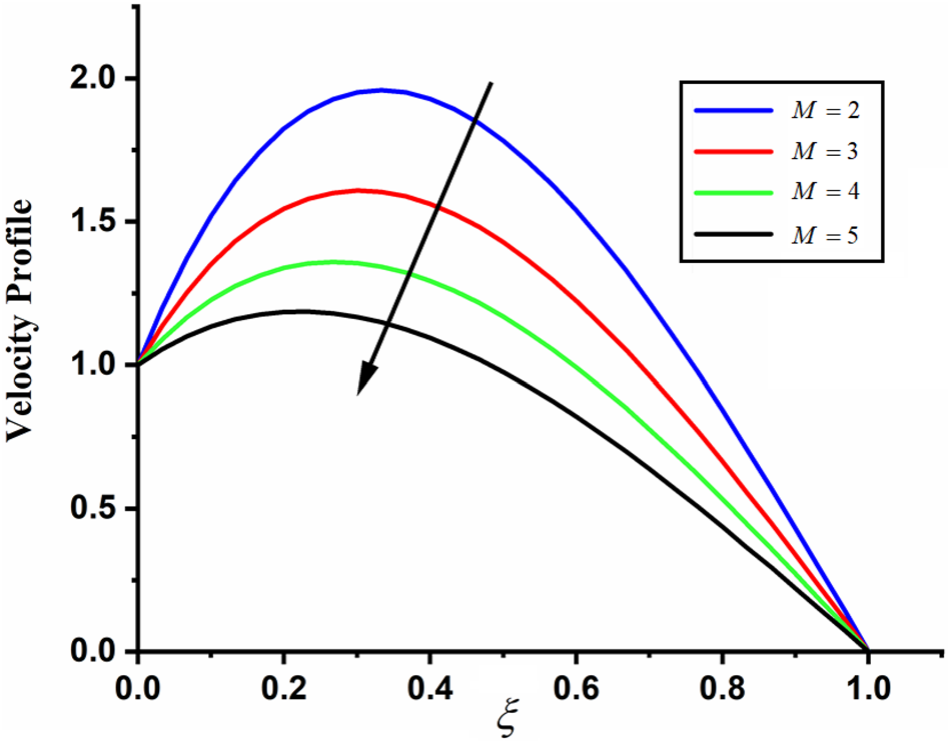
The impact of $M$ on fractal-fractional couple stress fluid velocity in channel when, $\alpha = 0.5$, $\beta = 0.5$, $Gr = 2$, $Gm = 3$, $K = 3$, $\eta_{A} = 1$, $P = 1.5$, $\Pr = 1.5$, ${\text{Re}} = 2.5$ and $Sc = 0.3$.

**Figure 8:**
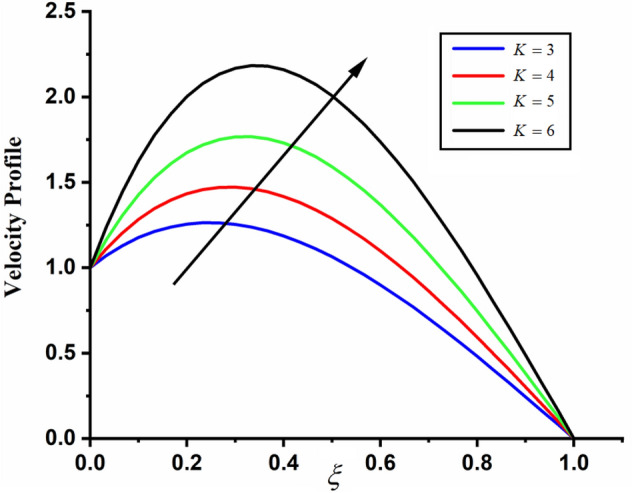
The impact of $K$ on fractal-fractional couple stress fluid velocity in channel when, $\alpha = 0.5$, $\beta = 0.5$, $Gr = 2$, $Gm = 3$, $M = 2$, $\eta_{A} = 1$, $P = 1.5$, $\Pr = 1.5$, ${\text{Re}} = 2.5$ and $Sc = 0.3$.

Figure 1 shows the physical sketch of the proposed problem. The effect of fractal dimension $\beta$ on velocity profile is highlighted in Fig. 2. From the graph it is quite clear that for greater values of fractal parameter result a decay in the fluid velocity it is due to the power law kernel. The effect of fractional parameter $\alpha$ is shown in Fig. 3. From the figure a decreasing in the CSF velocity is noticed. The influence of $\alpha$ on CSF velocity is similar to the effect of fractal dimension $\beta$ on the velocity field. The comparison between fractal-fractional CSF velocity and fractional velocity is plotted in Fig. 4. From the figure it can be noticed that the magnitude of fractional velocity is greater than fractal-fractional velocity. In this paper we have added a parameter $\beta$ known as fractal-fractional dimension. This parameter $\beta$ shows the combined effect of fractal-fractional derivative with fractional derivative. By introducing $\beta$ in CSF flow can explain better memory effect compared to fractional and classical CSF fluid. The effect of Gr and Gm on CSF velocity is highlighted in Figs. 5 and 6 respectively. From the graphs it is very clear that for greater values of Gr and Gm result an increase in the CSF velocity. This increase in the fluid velocity is due to the fact that Gr and Gm are the ratios of boyancy forces and viscous forces which are responsible to accelerate the fluid motion as a result CSF velocity increases. The effect of magnetic parameter $M$ is highlighted if Fig. 7. From the figure it is clear that increasing $M$ result a decrease in the fractal-fractional CSF velocity. This is due to the fact that for greater values of $M$ Lorentz forces increases in the CSF which control the boundary layer thickness as a result velocity of the fractal-fractional CSF decreases. The influence of porosity $K$ on fractal-fractional CSF velocity is highlighted in Fig. 8. From this figure it seems that the CSF velocity increases with the greater values of $K$ it is due to the fact that increasing $K$ increases the pores in the channel as a result the fluid velocity accelerates.

**Figure 9:**
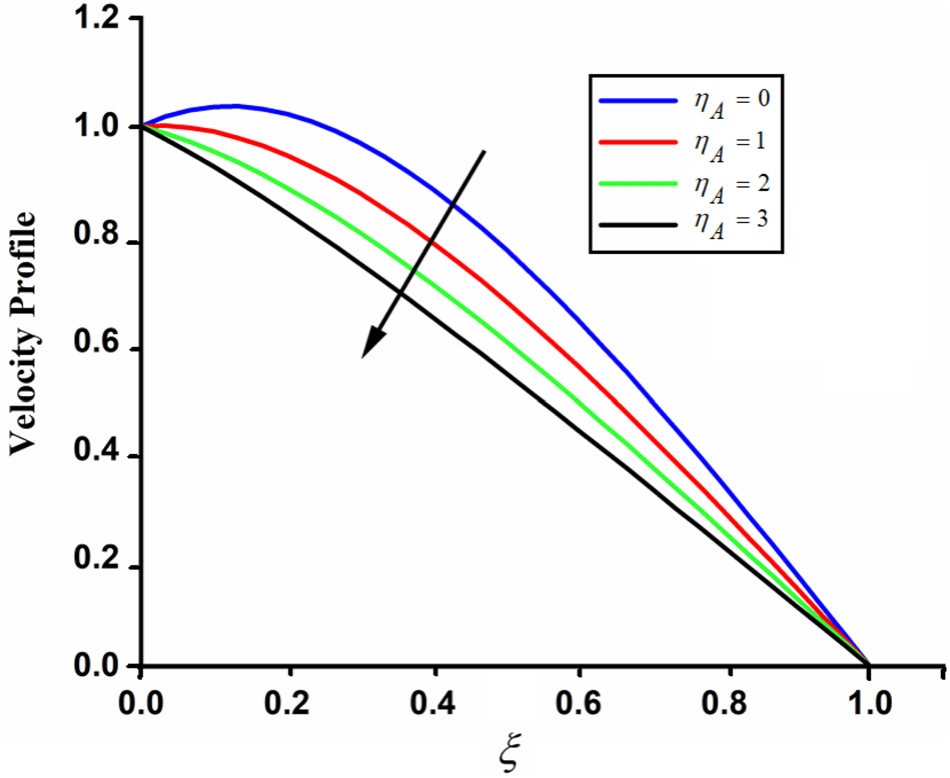
The impact of $\eta_{A}$ on fractal-fractional couple stress fluid velocity in channel when, $\alpha = 0.5$, $\beta = 0.5$, $Gr = 2$, $Gm = 3$, $M = 2$, $K = 3$, $P = 1.5$, $\Pr = 1.5$, ${\text{Re}} = 2.5$ and $Sc = 0.3$.

**Figure 10:**
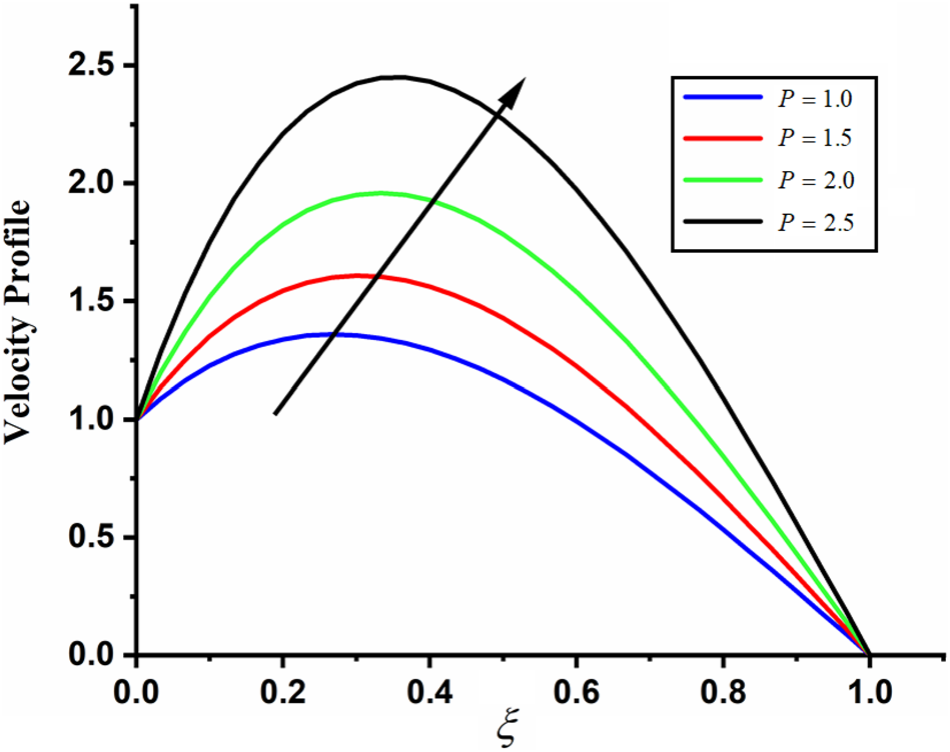
The impact of external pressure $P$ on fractal-fractional couple stress fluid velocity in channel when, $\alpha = 0.5$, $\beta = 0.5$, $Gr = 2$, $Gm = 3$, $M = 2$, $K = 3$, $\eta_{A} = 1$, $\Pr = 1.5$, ${\text{Re}} = 2.5$ and $Sc = 0.3$.

The effect of couple stress parameter $\eta_{A}$ is depicted in Fig. 9. This figure shows the influence of $\eta_{A}$ on the fractal-fractional velocity in channel. Increasing the values of couple stress parameter $\eta_{A}$ increases the viscosity of the fluid as a result the CSF velocity retards in channel. From this figure it can also be noticed that for $\eta_{A} = 0$ shows the comparison of simple Newtonian viscous fluid with fractal-fractional velocity in channel. The influence of external pressure $P$ is highlighted in Fig. 10. From the figure it can be noticed that increasing the values of $P$ result an increase in the CSF velocity in channel.

**Figure 11:**
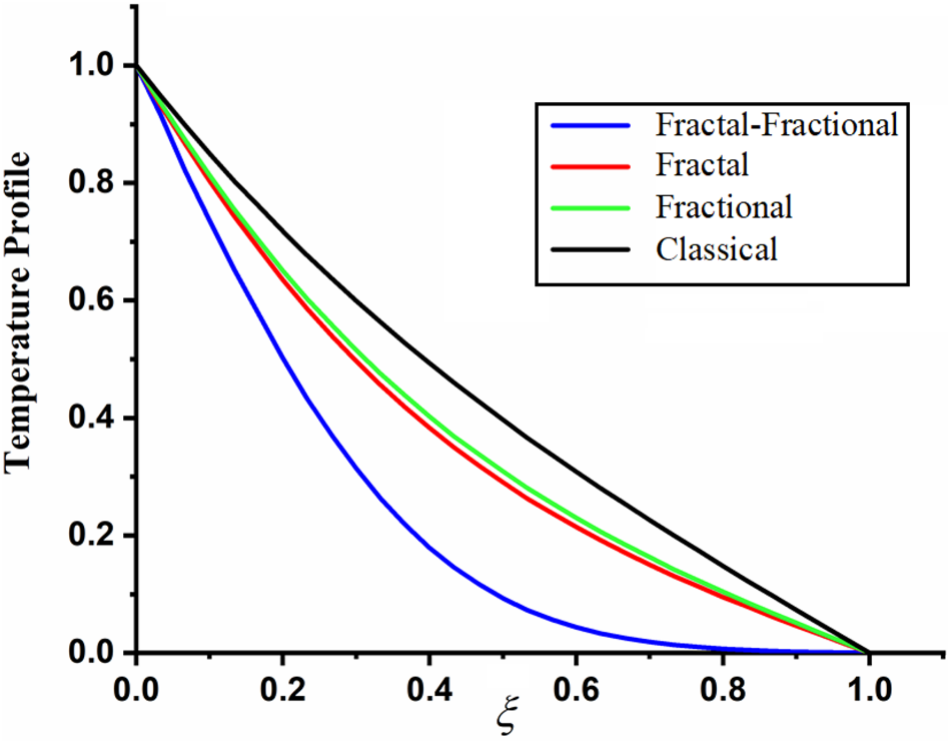
The comparison between fractal-fractional, fractal ,fractional and classical temperature when $\alpha = 0.5$ , $\beta = 0.5$, ${\text{Re}} = 3.2$, $t = 0.2$ and $\Pr = 0.5$.

**Figure 12:**
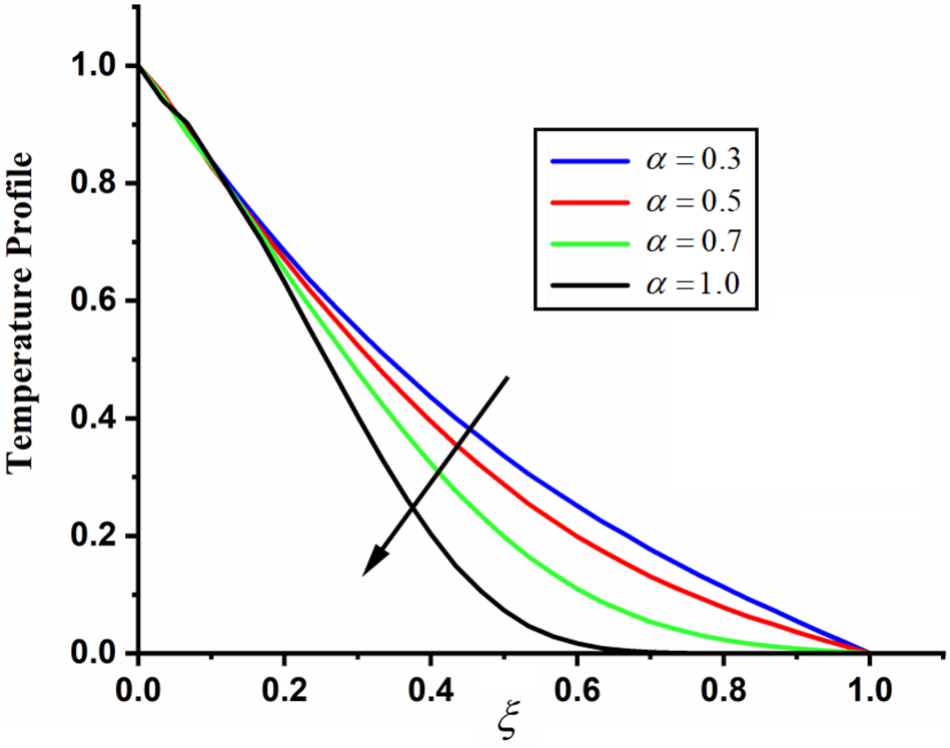
The impact of $\alpha$ on the temperature of couple stress fluid when, $\beta = 0.5$, ${\text{Re}} = 3.2$, $t = 0.2$ and $\Pr = 0.5$.

**Figure 13:**
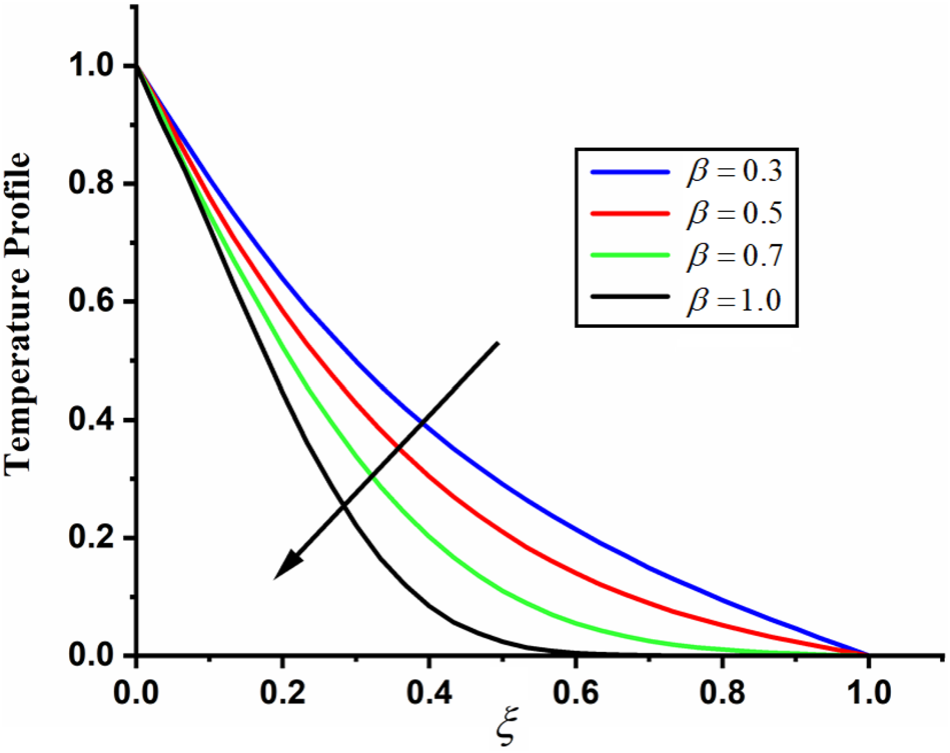
The impact of $\beta$ on the temperature of couple stress fluid when, $\alpha = 0.5$, ${\text{Re}} = 3.2$, $t = 0.2$ and $\Pr = 0.5$.

**Figure 14:**
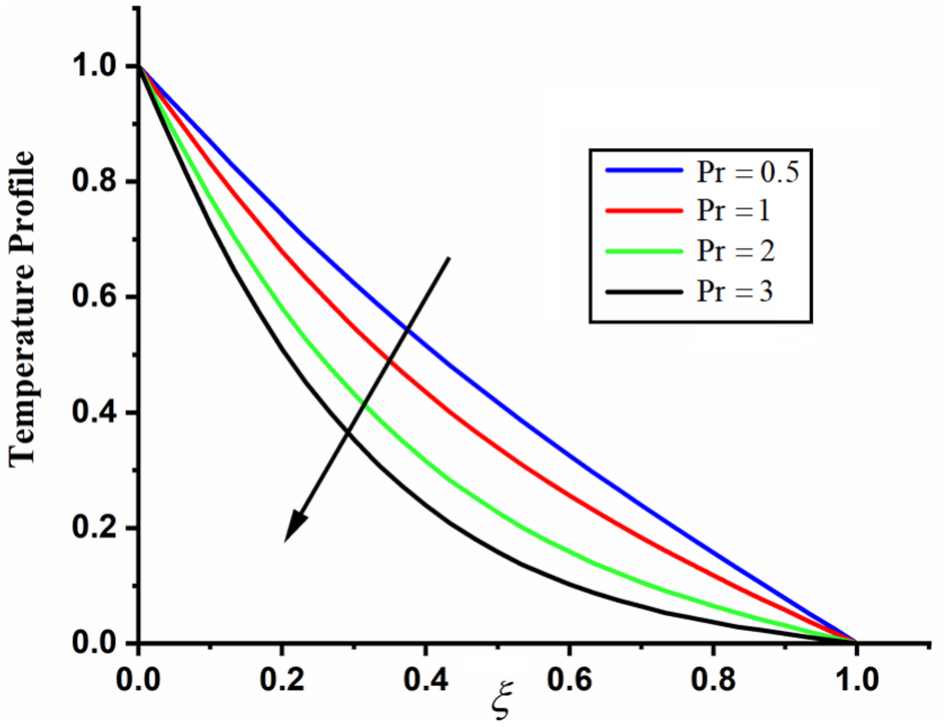
The impact of $\Pr$ on the temperature of couple stress fluid when,$\alpha = 0.3$, $\beta = 0.3$, ${\text{Re}} = 4$ and $t = 0.7$.

**Figure 15:**
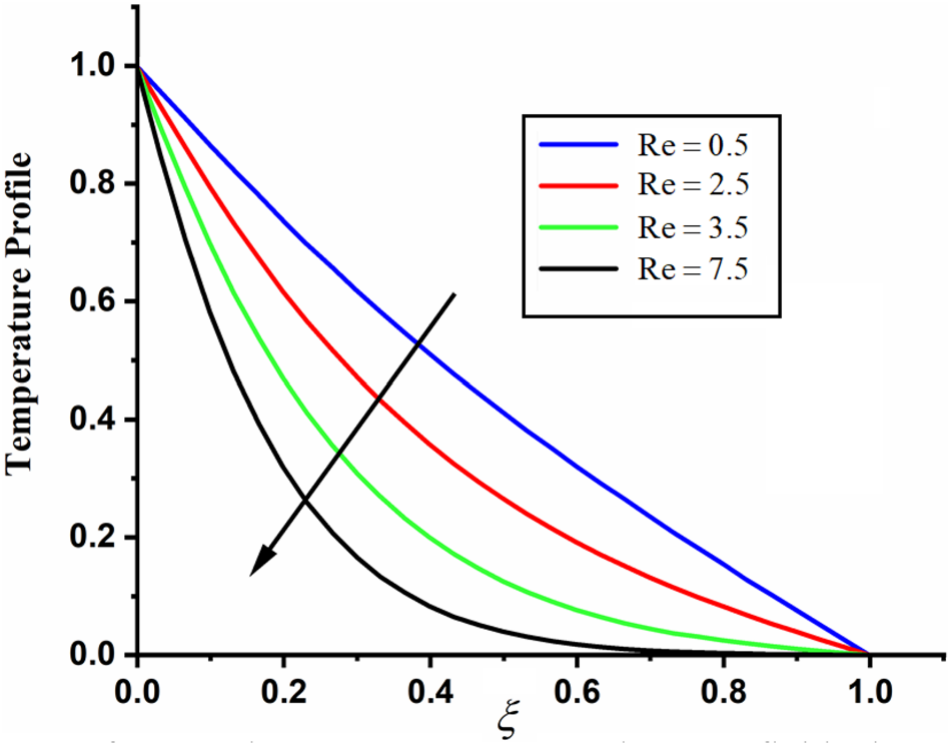
The impact of ${\text{Re}}$ on the temperature of couple stress fluid when $\alpha = 0.3$, $\beta = 0.3$, $t = 0.2$ and $\Pr = 2$.

The comparison of fractal-fractional temperature with fractal temperature $\beta$ and fractional temperature $\alpha$ is highlighted in Fig. 11. From the comparison we can see that the classical temperature is higher than fractal-fractional, fractal and fractional temperature. The influence of fractional parameter $\alpha$ and fractal parameter $\beta$ on temperature is depicted in Figs. 12 and 13 respectively. From both the figures it can be noticed that for the greater values of fractional parameter $\alpha$ and fractal parameter $\beta$ the temeprature of the CSF in channel reduces. The effect of $\Pr$ on temperature profile is highlighted in Fig. 14. From the figure we can observe that for larger values of $\Pr$ the CSF temperature decreases it is due to the fact that increasing $\Pr$ results a decrease in thermal conductivity of the fluid as a result the temperature of the fluid decreases. The influence of Reynolds umber on temeprature profile is shown in Fig. 15. From the figure it can be observed that greater values of Reynolds number decrease the temperature of CSF in channel.

**Figure 16:**
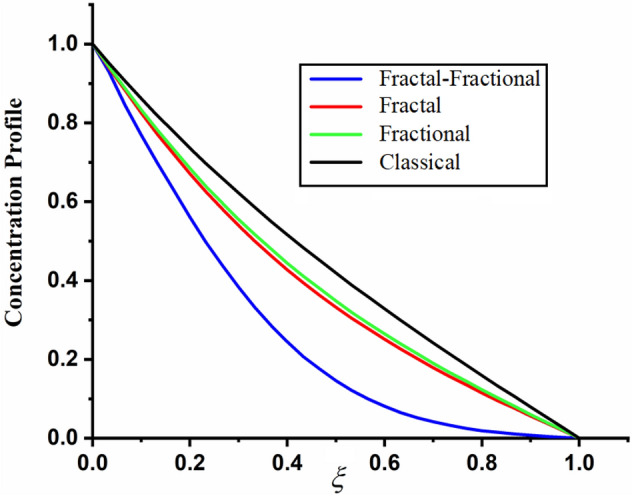
The comparison between fractal-fractional, fractal, fractional and classical concentration when $\alpha = 0.5$, $\beta = 0.5$, $t = 0.2$, $Sc = 0.5$ and ${\text{Re}} = 3$.

**Figure 17:**
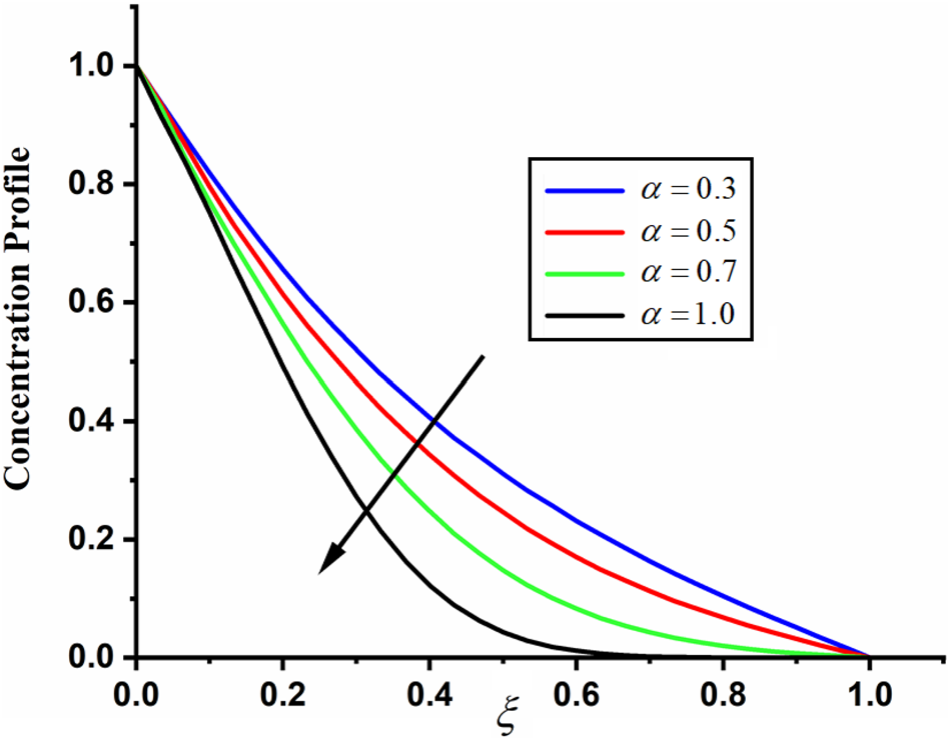
The impact of $\alpha$ on couple stress fluid concentration profile when,$\beta = 0.5$, $t = 0.2$, $Sc = 0.5$ and ${\text{Re}} = 3$.

**Figure 18:**
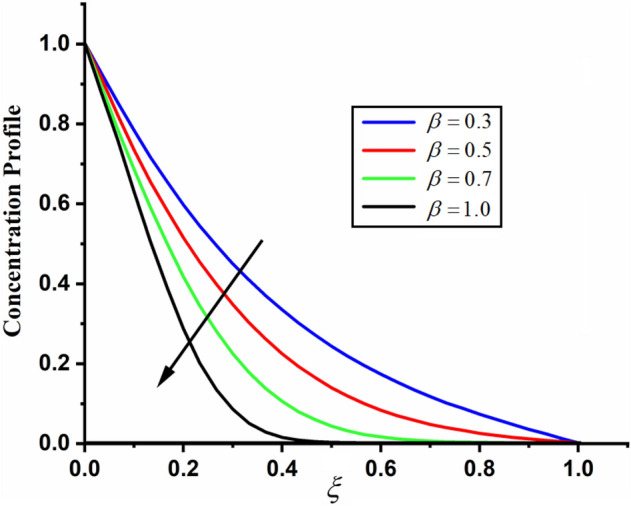
The impact of $\beta$ on couple stress fluid concentration profile when, $\alpha = 0.5$, $t = 0.2$, $Sc = 0.5$ and ${\text{Re}} = 3$.

**Figure 19:**
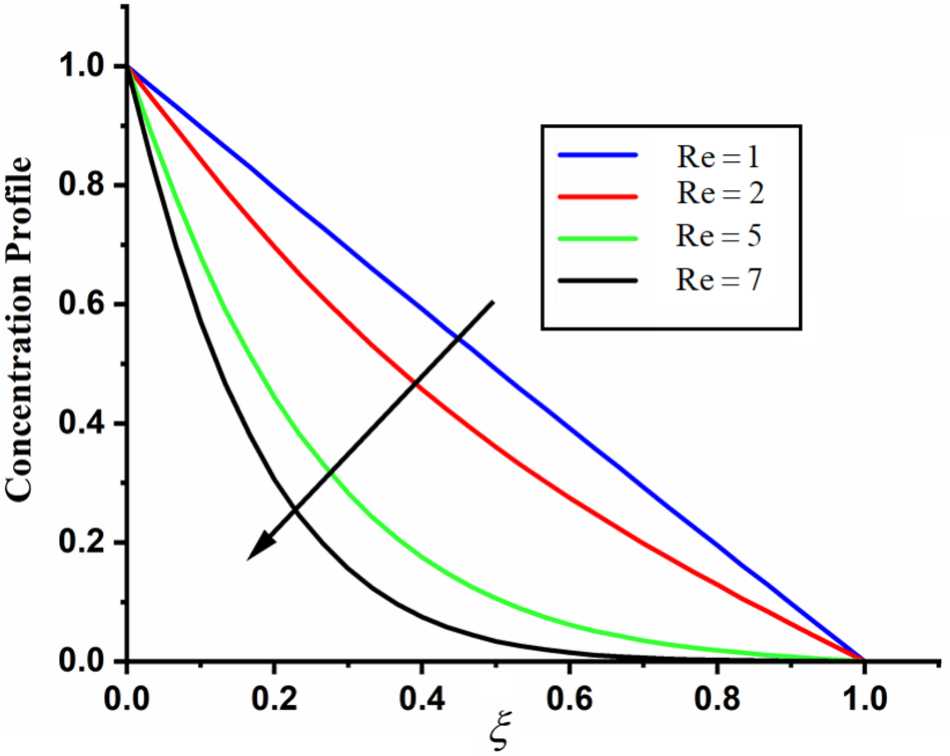
The impact of ${\text{Re}}$ on couple stress fluid concentration profile when $\alpha = 0.5$, $\beta = 0.5$, $t = 0.2$ and $Sc = 0.5$.

**Figure 20:**
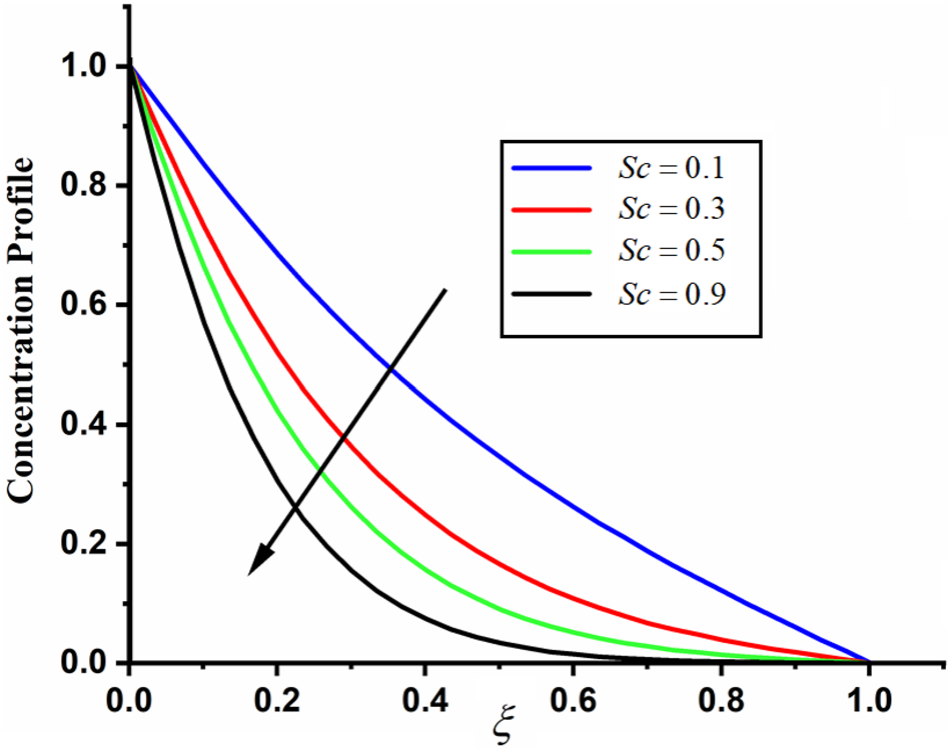
The impact of $Sc$ on couple stress fluid concentration profile when, $\alpha = 0.5$, $\beta = 0.5$, $t = 0.2$ and ${\text{Re}} = 3$.

**Figure 21:**
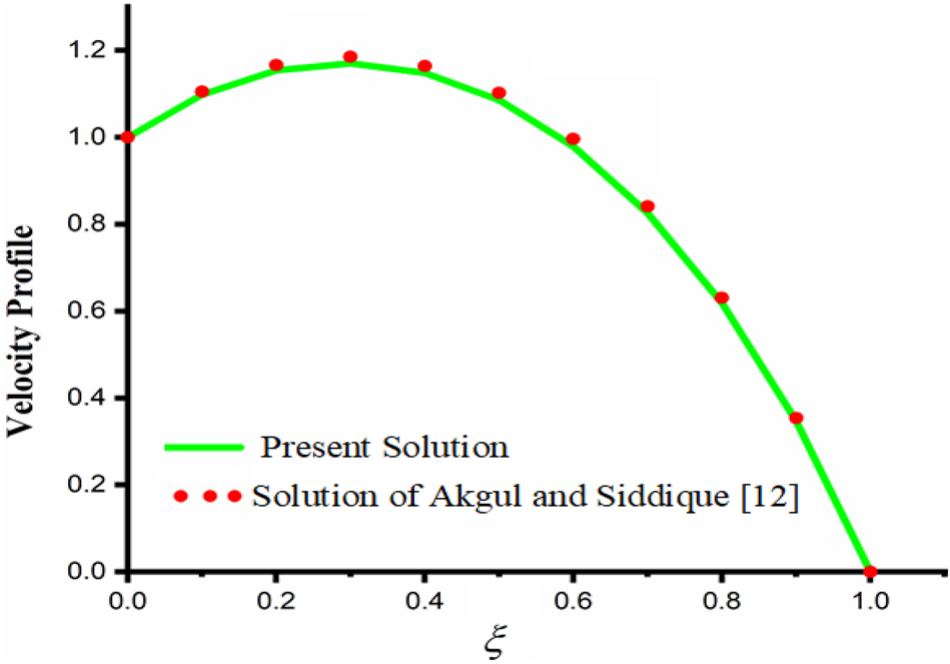
Comparison of the present solution to solution of Akgul and Siddique^12^ when, $\alpha = 0.5$, $\beta = 0.5$, $Gr = 0$, $Gm = 0$, $M = 2$, $\frac{1}{K} \to 0$, $\eta_{A} = 1$, $P = 0$, $\Pr = 1.5$, ${\text{Re}} = 2.5$ and $Sc = 0.3$.

The comparison of fractal-fractional, fractal and fractional concentration is highlighted in Fig. 16. From the figure one can noticed that the magnitude of classical concentration is higher than the concentration for fractal and fractional concentration. The effect of fractional parameter $\alpha$ and fractal parameter $\beta$ on concentration profile is highlighted in Figs. 17 and 18 respectively. From both the figures it can be noticed that for the greater values of fractional parameter $\alpha$ and fractal parameter $\beta$ in both the cases the concentration profile of the CSF in channel reduces. The influence of Reynolds number ${\text{Re}}$ on concentration profile is highlighted in Fig. 19. From this figure the greater values of Reynolds number decreases the concentration of the CSF in channel it is due to the fact that greater Reynolds number decreases the CSF motion as a result the concentration of the fluid decreases. The effect of Schmidth number Sc on concentration profile is highlighted in Fig. 20. Form the figure it can be notice that increasing the values of Sc result a decrease in the CSF concentration profile. The comparison between the present solution to the already published result is highlighted in Fig. 21. From the figure it can be seen that by putting (*Gr* = 0), (*Gm* = 0), *P* = 0 and $\frac{1}{K} \to 0$ our solutions reduced to the solutions recently obtained by Akgul and Siddique^12^ which verify our obtained solutions.

The skin friction for left and right plate are evaluated and presented in Tables 1 and 2 respectively. From these tables skin friction variation can be noticed for varying different parameters. The bold values in the tables show the change in skin friction in that specific parameter. Similarly, Table 3 shows the Nusselt number variation for different parameters. Table 4 shows the variation in Sherwood number for different parameters. In the Tables 1, 2, 3 and 4 bold values represents the changes in the specific parameter and its effect on the ski friction, Nusselt number and Sherwood number.

**Table 1:** The skin friction of couple stress fluid at left plate.

$\alpha$	$\beta$	$\tau$	$Gr$	$Gm$	$\Pr$	$Sc$	$M$	$K$	$\eta_{A}$	$P$	${\text{Re}}$	$Sf_{lp}$
0.4	0.4	1.2	10	8	14	2.5	5	1.5	2.2	3	2.5	2.0631
**0.5**	0.4	1.2	10	8	14	2.5	5	1.5	2.2	3	2.5	1.9375
0.4	**0.5**	1.2	10	8	14	2.5	5	1.5	2.2	3	2.5	1.8402
0.4	0.4	**2**	10	8	14	2.5	5	1.5	2.2	3	2.5	3.821
0.4	0.4	1.2	**15**	8	14	2.5	5	1.5	2.2	3	2.5	4.0592
0.4	0.4	1.2	10	**12**	14	2.5	5	1.5	2.2	3	2.5	3.8743
0.4	0.4	1.2	10	8	**20**	2.5	5	1.5	2.2	3	2.5	1.2751
0.4	0.4	1.2	10	8	14	**3**	5	1.5	2.2	3	2.5	1.9281
0.4	0.4	1.2	10	8	14	2.5	**6**	1.5	2.2	3	2.5	2.0321
0.4	0.4	1.2	10	8	14	2.5	5	**2**	2.2	3	2.5	2.0582
0.4	0.4	1.2	10	8	14	2.5	5	1.5	**3**	3	2.5	1.8573
0.4	0.4	1.2	10	8	14	2.5	5	1.5	2.2	**4**	2.5	2.0825
0.4	0.4	1.2	10	8	14	2.5	5	1.5	2.2	3	**3.5**	2.7352

**Table 2:** The skin friction of couple stress fluid at right plate:

$\alpha$	$\beta$	$\tau$	$Gr$	$Gm$	$\Pr$	$Sc$	$M$	$K$	$\eta_{A}$	$P$	${\text{Re}}$	$Sf_{lp}$
0.4	0.4	1.2	10	8	14	2.5	5	1.5	2.2	3	2.5	0.8083
**0.5**	0.4	1.2	10	8	14	2.5	5	1.5	2.2	3	2.5	0.6352
0.4	**0.5**	1.2	10	8	14	2.5	5	1.5	2.2	3	2.5	0.5873
0.4	0.4	**2**	10	8	14	2.5	5	1.5	2.2	3	2.5	1.2352
0.4	0.4	1.2	**15**	8	14	2.5	5	1.5	2.2	3	2.5	1.6201
0.4	0.4	1.2	10	**12**	14	2.5	5	1.5	2.2	3	2.5	1.1401
0.4	0.4	1.2	10	8	**20**	2.5	5	1.5	2.2	3	2.5	0.6731
0.4	0.4	1.2	10	8	14	**3**	5	1.5	2.2	3	2.5	0.7031
0.4	0.4	1.2	10	8	14	2.5	**6**	1.5	2.2	3	2.5	0.7481
0.4	0.4	1.2	10	8	14	2.5	5	**2**	2.2	3	2.5	0.9202
0.4	0.4	1.2	10	8	14	2.5	5	1.5	**3**	3	2.5	0.5375
0.4	0.4	1.2	10	8	14	2.5	5	1.5	2.2	**4**	2.5	0.8821
0.4	0.4	1.2	10	8	14	2.5	5	1.5	2.2	3	**3.5**	0.9383

**Table 3:** Nusselt number variation against different parameters.

$\alpha$	$\beta$	$\tau$	$\Pr$	${\text{Re}}$	$Nu_{Left\,\,Plate}$
0.5	0.5	1	14	2.5	1.511
**0.7**	0.5	1	14	2.5	1.06
0.5	**0.7**	1	14	2.5	1.061
0.5	0.5	**1.5**	14	2.5	1.424
0.5	0.5	1	**16**	2.5	1.848
0.5	0.5	1	14	**3**	1.654

**Table 4:** Sherwood number variation against different parameters.

$\alpha$	$\beta$	$\tau$	$Sc$	${\text{Re}}$	$Sh_{Left\,\,Plate}$
0.5	0.5	1.2	0.8	2.2	0.9
**0.7**	0.5	1.2	0.8	2.2	0.719
0.5	**0.7**	1.2	0.8	2.2	0.493
0.5	0.5	**1.5**	0.8	2.2	0.894
0.5	0.5	1.2	**1.2**	2.2	0.938
0.5	0.5	1.2	0.8	**3**	0.925

## Conclusion

The present paper is focused to study the applications of fractal and fractional derivative on the unsteady MHD CSF in channel with power law kernel. The fractal-fractional CSF is assumed to flow in channel embedded in porous medium. The unsteady CSF with heat and mass transfer passes through the channel in the presence of external pressure. The implicit finite difference method is applied to obtain the numerical solutions of the proposed fractal-fractional CSF model of generalized Couette flow. During the analysis of the present study the following results are obtaiend.

- The velocity, temperature and concentration of the magnetohydrodynamics CSF in porous channel decreases for the greater values of both fractional parameter $\alpha$ and fractal parameter $\beta$ respectively.
- In the fluid dynamics the fractal-fractional model of CSF explain good memory effect as compared to fractional model of CSF.
- CSF velocity in porous channel decrease with the increase in $\alpha$,$\beta$,$M$ and $\eta_{A}$ .
- CSF velocity in porous channel increases with the higher values of $Gr$,$Gm$ and $K$.
- The temperature of the CSF is decreases with the higher values of $$\alpha ,$$$$\beta$$,$$\Pr$$ and $${\text{Re}}$$.
- The concentration of the CSF is decreases with the higher values of $$\alpha ,$$$$\beta$$,$$Sc$$ and $${\text{Re}}$$.

## Data Availability

Data is available upon reasonable request to corresponding author.

## References

[1] Tenreiro Machado JA, Silva MF, Barbosa RS, Jesus IS, Reis CM, Marcos MG, Galhano AF (2010). Some applications of fractional calculus in engineering. Math. Probl. Eng.

[2] Dalir M, Bashour M (2010). Applications of fractional calculus. Appl. Math. Sci.

[3] Tavazoei MS, Haeri M, Jafari S, Bolouki S, Siami M (2008). Some applications of fractional calculus in suppression of chaotic oscillations. IIEEE Trans. Ind. Electron.

[4] Sabatier, J. A. T. M. J., Agrawal, O. P., & Machado, J. T. Fract. Calc. Appl. Anal, (Vol**. 4**, No. 9). Dordrecht: Springer, (2007).

[5] Koca I, Atangana A (2017). Solutions of Cattaneo-Hristov model of elastic heat diffusion with Caputo-Fabrizio and Atangana-Baleanu fractional derivatives. J. Therm. Sci.

[6] Atangana, A., & Baleanu, D. New fractional derivatives with nonlocal and non-singular kernel: theory and application to heat transfer model. *arXiv preprint *arXiv: 1602.03408, (2016).

[7] Arif M, Ali F, Sheikh NA, Khan I, Nisar KS (2019). Fractional model of couple stress fluid for generalized Couette flow: A comparative analysis of Atangana-Baleanu and Caputo-Fabrizio fractional derivatives. IEEE Access.

[8] Podlubny I (1998). Fractional Differential Equations: An Introduction to Fractional Derivatives, Fractional Differential Equations, to Methods of Their Solution and Some of Their Applications.

[9] Abdeljawad T, Baleanu D (2018). On fractional derivatives with generalized Mittag-Leffler kernels. Adv. Differ. Equ..

[10] Atangana A (2017). Fractal-fractional differentiation and integration: Connecting fractal calculus and fractional calculus to predict complex system. Chaos Solitons Fractals.

[11] Ali Z, Rabiei F, Shah K, Abdul Majid Z (2021). Dynamics of SIR mathematical model for COVID-19 outbreak in Pakistan under Fractal-fractional derivative. Fractals.

[12] Akgül A, Siddique I (2021). Novel applications of the magnetohydrodynamics couple stress fluid flows between two plates with fractal-fractional derivatives. Numer. Methods Partial Differ. Equ..

[13] Esmonde H (2020). Fractal and fractional derivative modelling of material phase change. Fractal Fract..

[14] Akgül, A. Analysis and new applications of fractal fractional differential equations with power law kernel. *Discrete & Continuous Dynamical Systems-S*, (2020).

[15] Muzychka YS, Edge J (2008). Laminar non-Newtonian fluid flow in noncircular ducts and microchannels. J. Fluids Eng.

[16] Chhabra RP, Richardson JF (1999). Non-Newtonian flow in the process industries: fundamentals and engineering applications.

[17] Pawar SS, Sunnapwar VK (2013). Experimental studies on heat transfer to Newtonian and non-Newtonian fluids in helical coils with laminar and turbulent flow. Exp. Therm. Fluid Sci.

[18] Hoyt JW (1999). Some applications of non-Newtonian fluid flow. Rheology Series.

[19] Sohail M, Naz R, Shah Z, Kumam P, Thounthong P (2019). Exploration of temperature dependent thermophysical characteristics of yield exhibiting non-Newtonian fluid flow under gyrotactic microorganisms. AIP Adv..

[20] Bao, K., Lavrov, A., & Nilsen, H. M. Numerical Modelling of Non-newtonian Fluid Flow in Fractures and Porous Media. In ECMOR XV-15th European Conference on the Mathematics of Oil Recovery (pp. cp-494). *European Association of Geoscientists & Engineers*, (2016, August).

[21] Gowda RP, Kumar RN, Aldalbahi A, Issakhov A, Prasannakumara BC, Rahimi-Gorji M, Rahaman M (2021). Thermophoretic particle deposition in time-dependent flow of hybrid nanofluid over rotating and vertically upward/downward moving disk. Surf. Interfaces.

[22] Gowda RP, Rauf A, Kumar RN, Prasannakumara BC, Shehzad SA (2021). Slip flow of Casson-Maxwell nanofluid confined through stretchable disks. Indian J. Phys.

[23] Zhou SS, Khan MI, Qayyum S, Prasannakumara BC, Kumar RN, Khan SU, Chu YM (2021). Nonlinear mixed convective Williamson nanofluid flow with the suspension of gyrotactic microorganisms. Int. J. Mod. Phys. B.

[24] Stokes VK (1984). Couple stresses in fluids. Theories of Fluids with Microstructure.

[25] Arif M, Ali F, Khan I, Nisar KS (2020). A time fractional model with non-singular kernel the generalized Couette flow of couple stress nanofluid. IEEE Access.

[26] Adesanya SO, Souayeh B, Rahimi-Gorji M, Khan MN, Adeyemi OG (2019). Heat irreversibiility analysis for a couple stress fluid flow in an inclined channel with isothermal boundaries. J. Taiwan Inst Chem Eng.

[27] Krishna MV, Chamkha AJ (2019). MHD peristaltic rotating flow of a couple stress fluid through a porous medium with wall and slip effects. Spec. Top. Rev. Porous Media.

[28] Khan NA, Mahmood A, Ara A (2013). Approximate solution of couple stress fluid with expanding or contracting porous channel. Eng. Comput..

[29] Akhtar S (2016). Flows between two parallel plates of couple stress fluids with time-fractional Caputo and Caputo-Fabrizio derivatives. Eur. Phys. J. Plus.

[30] Kumar RN, Gowda RP, Abusorrah AM, Mahrous YM, Abu-Hamdeh NH, Issakhov A, Prasannakumara BC (2021). Impact of magnetic dipole on ferromagnetic hybrid nanofluid flow over a stretching cylinder. Phys. Scr.

[31] Gowda RP, Kumar RN, Rauf A, Prasannakumara BC, Shehzad SA (2021). Magnetized flow of sutterby nanofluid through cattaneo-christov theory of heat diffusion and stefan blowing condition. Appl. Nanosci.

[32] Gowda RP, Kumar RN, Prasannakumara BC, Nagaraja B, Gireesha BJ (2021). Exploring magnetic dipole contribution on ferromagnetic nanofluid flow over a stretching sheet: An application of Stefan blowing. J. Mol. Liq.

[33] Yusuf TA, Mabood F, Prasannakumara BC, Sarris IE (2021). Magneto-bioconvection flow of williamson nanofluid over an inclined plate with gyrotactic microorganisms and entropy generation. Fluids.

[34] Kumar RN, Jyothi AM, Alhumade H, Gowda RP, Alam MM, Ahmad I, Prasannakumara BC (2021). Impact of magnetic dipole on thermophoretic particle deposition in the flow of Maxwell fluid over a stretching sheet. J. Mol. Liq.

[35] Naveen Kumar, R., Punith Gowda, R. J., Prasanna, G. D., Prasannakumara, B. C., Nisar, K. S., & Jamshed, W. Comprehensive study of thermophoretic diffusion deposition velocity effect on heat and mass transfer of ferromagnetic fluid flow along a stretching cylinder. *P I Mech Eng E-J Pro*, 09544089211005291, (2021).

[36] Punith Gowda RJ, Naveen Kumar R, Jyothi AM, Prasannakumara BC, Sarris IE (2021). Impact of binary chemical reaction and activation energy on heat and mass transfer of marangoni driven boundary layer flow of a non-Newtonian nanofluid. Processes.

[37] Gowda RP, Al-Mubaddel FS, Kumar RN, Prasannakumara BC, Issakhov A, Rahimi-Gorji M, Al-Turki YA (2021). Computational modelling of nanofluid flow over a curved stretching sheet using Koo-Kleinstreuer and Li (KKL) correlation and modified Fourier heat flux model. Chaos, Solitons Fractals.

[38] Li YX, Khan MI, Gowda RP, Ali A, Farooq S, Chu YM, Khan SU (2021). Dynamics of aluminum oxide and copper hybrid nanofluid in nonlinear mixed Marangoni convective flow with entropy generation: Applications to renewable energy. Chin. J. Phys.

[39] Xiong PY, Hamid A, Chu YM, Khan MI, Gowda RP, Kumar RN, Qayyum S (2021). Dynamics of multiple solutions of Darcy-Forchheimer saturated flow of Cross nanofluid by a vertical thin needle point. Eur. Phys. J. Plus.

[40] Ali F, Ahmad Z, Arif M, Khan I, Nisar KS (2020). A time fractional model of generalized Couette flow of couple stress nanofluid with heat and mass transfer: Applications in engine oil. IEEE Access.

